# Synthesis and Structural Characterization of *p*-Carboranylamidine Derivatives †

**DOI:** 10.3390/molecules28093837

**Published:** 2023-04-30

**Authors:** Nicole Harmgarth, Phil Liebing, Volker Lorenz, Felix Engelhardt, Liane Hilfert, Sabine Busse, Rüdiger Goldhahn, Frank T. Edelmann

**Affiliations:** 1 Chemisches Institut, Otto-von-Guericke-Universität Magdeburg, Universitätsplatz 2, 39106 Magdeburg, Germanyvolker.lorenz@ovgu.de (V.L.); liane.hilfert@ovgu.de (L.H.); sabine.busse@ovgu.de (S.B.); 2 Institut für Anorganische und Analytische Chemie, Friedrich-Schiller-Universität Jena, Humboldtstr. 8, 07743 Jena, Germany; phil.liebing@uni-jena.de; 3 Institut für Physik, Otto-von-Guericke-Universität Magdeburg, Universitätsplatz 2, 39106 Magdeburg, Germany; ruediger.goldhahn@ovgu.de

**Keywords:** boron, carborane, *p*-carborane, lithiation, carboranylamidinate

## Abstract

In this contribution, the first amidinate and amidine derivatives of *p*-carborane are described. Double lithiation of *p*-carborane (**1**) with *n*-butyllithium followed by treatment with 1,3-diorganocarbodiimides, R–N=C=N–R (R = *^i^*Pr, Cy (= cyclohexyl)), in DME or THF afforded the new *p*-carboranylamidinate salts *p*-C_2_H_10_B_10_[C(N*^i^*Pr)_2_Li(DME)]_2_ (**2**) and *p*-C_2_H_10_B_10_[C(NCy)_2_Li(THF)_2_]_2_ (**3**). Subsequent treatment of **2** and **3** with 2 equiv. of chlorotrimethylsilane (Me_3_SiCl) provided the silylated neutral bis(amidine) derivatives *p*-C_2_H_10_B_10_[C{*^i^*PrN(SiMe_3_)}(=N*^i^*Pr)]_2_ (**4**) and *p*-C_2_H_10_B_10_[C{CyN(SiMe_3_)}(=NCy)]_2_ (**5**). The new compounds **3** and **4** have been structurally characterized by single-crystal X-ray diffraction. The lithium carboranylamidinate **3** comprises a rare trigonal planar coordination geometry around the lithium ions.

## 1. Introduction

**Figure 1:**
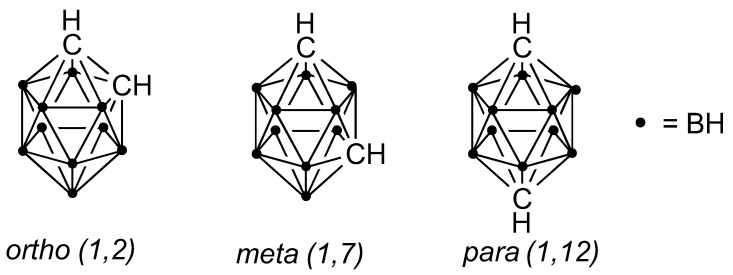
Three isomers of the icosahedral *closo*-carborane cage C_2_B_10_H_12_.

Icosahedral *closo*-carborane cage compounds of the general composition C_2_B_10_H_12_ can be viewed as 3D molecular analogs of benzene [1]. They are of high scientific and technological interest due to a variety of practical applications, e.g., in materials science [2,3,4,5,6,7,8,9,10,11,12,13], homogeneous catalysis [14,15,16,17,18,19,20,21], and medicinal chemistry [22,23,24,25,26,27,28,29]. Moreover, carborane derivatives are widely employed in coordination chemistry and as building blocks in supramolecular, bio-inorganic, and organometallic chemistry [30,31,32,33,34]. Depending on the position of the two carbon atoms in the carborane cage, three isomers can be distinguished. As shown in Figure 1, these are *ortho*-carborane (*o*-carborane, 1,2-C_2_B_10_H_12_), *meta*-carborane (*m*-carborane, 1,7-C_2_B_10_H_12_), and *para*-carborane (*p*-carborane, 1,12-C_2_B_10_H_12_) [35]. Most readily available among these is *o*-carborane, while the other two are made by thermal rearrangement of the *ortho*-isomer. This is why *p*-carborane is the most expensive precursor and its derivative chemistry is the least studied [35].

**Scheme 1:**
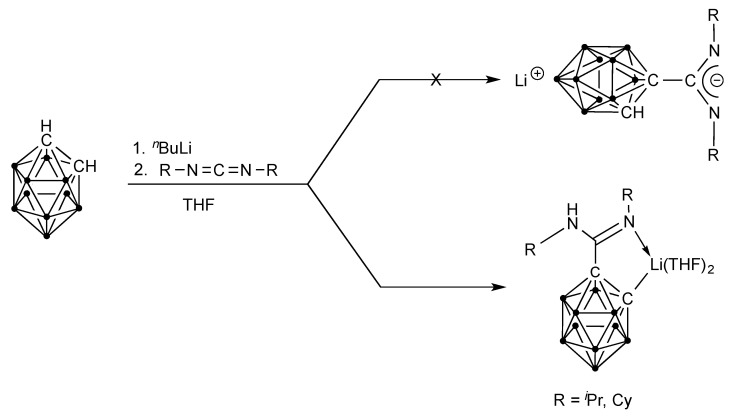
Preparation of lithium carboranylamidinates derived from *o*-carborane.

In 2010, we reported a novel type of *o*-carborane-based *N*-chelating ligands which were named carboranylamidinates. These were obtained in the form of their lithium derivatives by in situ lithiation of *o*-carborane using *n*-butyllithium followed by treatment with one equivalent of 1,3-diorganocarbodiimides, R–N=C=N–R (R = *^i^*Pr, Cy (= cyclohexyl)). As illustrated in Scheme 1, the resulting carboranylamidinate anions were quite unique as they combined the highly versatile amidinate ligand system, [RC(NR′)_2_]^−^ [36,37,38,39,40], with a σ-bond to the carborane cage. Subsequently, the lithium salts served as precursors for a variety of main-group and transition metal complexes comprising carboranylamidinate ligands [41,42,43,44,45]. In all these complexes, the carboranylamidinate ligands adopt the characteristic *κC*,*κN*-chelating coordination mode instead of the regular *κN*,*κN′*-chelating mode of metal-coordinated amidinate anions.

**Scheme 2:**
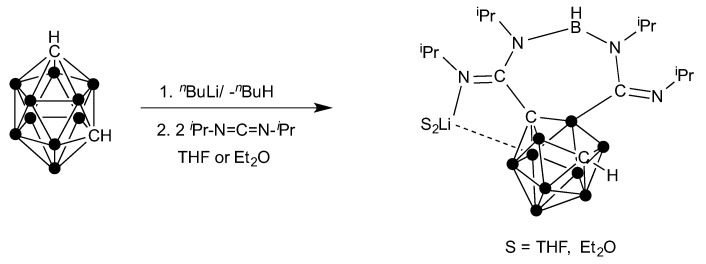
Formation of polycylic diazadiborepines from *m*-carborane.

Thus far, the formation of *κC*,*κN*-chelating carboranylamidinate anions has been limited to compounds derived from *o*-carborane. In 2014, we reported that similar reactions starting from *m*-carborane take a completely different course. As illustrated in Scheme 2, successive treatment of *m*-carborane with *n*-butyllithium and 1,3-di-*iso*-propylcarbodiimide did not lead to the formation of a related carboranylamidinate anion. Instead, an unprecedented deboronation reaction of the *m*-carborane took place, in which a BH group was detached from the carborane cage and incorporated into a *nido*-carborane-anellated diazadiborepine ring system. 1,3-dicyclohexylcarbodiimide reacted in a very similar manner but afforded a slightly modified seven-membered diazadiborepine ring system [46].

Until now, the question remained of how the third isomer, *p*-carborane (**1**), would behave in the same reaction sequence of lithiation and carbodiimide addition. Here, we present the answer to this question.

## 2. Results and Discussion

### 2.1. Synthesis and Characterization

**Scheme 3:**
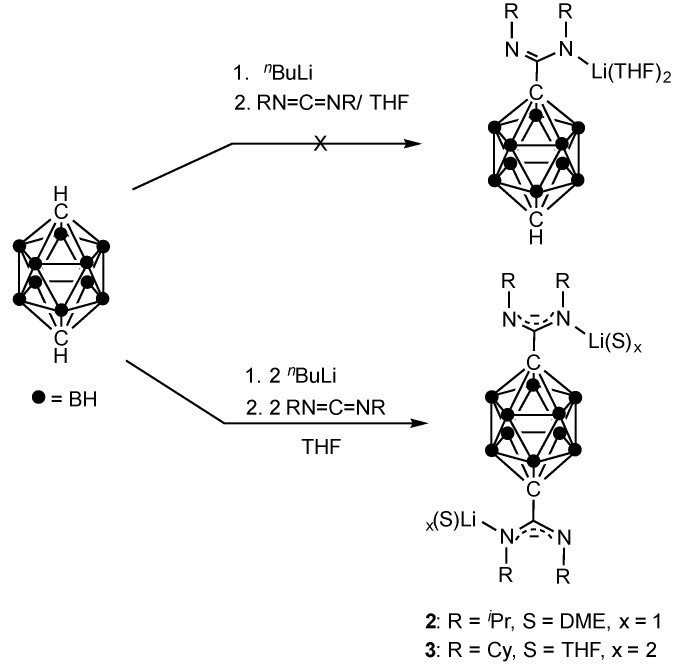
Synthetic route to the title compounds **2** and **3**.

**Scheme 4:**
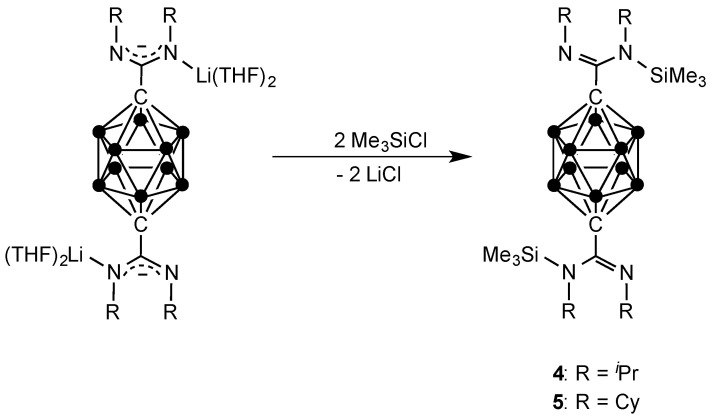
Preparation of the silylated bis(amidine) derivatives **4** and **5**.

In the first set of experiments, THF solutions of *p*-carborane were metalated with 1 equiv. of *n*-butyllithium and then treated in situ with two different carbodiimides R–N=C=N–R (R = *^i^*Pr, Cy). Under these conditions, only small amounts (ca. 20% yield) of crystalline products could be isolated, which were difficult to separate from unreacted *p*-carborane (NMR control). This finding implied that the envisaged *mono*-amidinate derivatives shown in the upper equation in Scheme 3 were not formed as pure reaction products and that disubstitution was instead the preferred reaction pathway. This assumption was soon confirmed by adjusting the stoichiometric ratio of the reactants to 1:2:2 according to the second equation in Scheme 3. Under these conditions, the new compounds **2** (R = *^i^*Pr) and **3** (R = Cy) could be isolated as pure crystalline solids in significantly improved yields of 51% (**2**) and 46% (**3**), respectively. Both lithium amidinate salts are readily soluble in THF, DME, and diethyl ether. Crystallization from DME (**2**) and THF (**3**) afforded the nicely crystalline solvates depicted in Scheme 4.

Both bis(anionic) title compounds **2** and **3** were fully characterized through the usual set of elemental analyses and spectroscopic methods. In the IR spectra, strong bands at 1523 cm^−1^ (**2**) and 1543 cm^−1^ (**3**) are typical for the stretching vibrations of the delocalized amidinate NCN units [36,37,38,39,40]. Medium strong bands in the range of 2590–2620 cm^−1^ could be assigned to the B–H stretching vibrations, while the ν_as_(C-O-C) bands of the coordinated solvents appear around 1050 cm^−1^ as medium or strong bands. The ^1^H and ^13^C NMR spectra show the typical signals of the *iso*-propyl and cyclohexyl substituents, which do not need to be discussed here in detail (cf., Experimental Section and Supplementary Materials). In the ^1^H NMR spectra, the B–H hydrogens give rise to broad multiplets extending over a range of ca. 1.5 ppm. The ^13^C NMR chemical shifts of the carbon atoms of the NCN groups are 155.2 ppm (**2**) and 154.0 ppm (**3**), respectively. A ^13^C resonance of the quaternary carbon atoms within the carborane cage could be detected only in the spectrum of **2** (δ 93.3 ppm). All cage boron atoms give rise to a single resonance around −14 ppm in the ^11^B NMR spectra of both amidinate salts. Apparently, the centrosymmetric structure leads to very similar chemical shifts of the boron atoms so that the signals could not be further resolved. Finally, ^7^Li NMR spectra displayed only one signal around 0.1 ppm. As expected for salt-like compounds, the mass spectra of **2** and **3** did not show the respective molecular ions but only fragment peaks of the unsolvated carboranylamidinate anions (cf., Experimental Section).

Remarkably, the formation of the lithium carboranylamidiate represents the first incidence of a “normal” reactivity of a lithiated carborane with carbodiimides. This means that 1,12-dilithiocarborane behaves toward carbodiimides like any other organolithium reagents and adds to the central carbon atom of the N=C=N moiety under the formation of regular amidinate anion of the type [RC(NR′)_2_]^−^ [36,37,38,39,40]. This finding reveals that all three C_2_B_10_H_12_ isomers behave differently in their reactivity toward 1,3-diorganocarbodiimides.

As an initial reactivity study involving the lithium carboranylamidinate salts **2** and **3**, we investigated silylation reactions with chlorotrimethylsilane, Me_3_SiCl, which should lead to the formation of neutral bis-silylated amidine derivatives, as illustrated in Scheme 4.

Both reactions were carried out in THF solutions at r.t. Work-up via extraction with toluene afforded the bis-silylated products **4** and **5** as colorless crystals in moderate yields (**4**: 54%, **5**: 43%). Both compounds dissolve freely in diethyl ether and toluene, and are moderately moisture-sensitive due to the presence of Si–N bonds. Besides an X-ray structural analysis of **4** (see next paragraph), all analytical and spectroscopic data were in excellent agreement with the formation of bis(silylated) *p*-carboranyl-bis(amidines). Highly characteristic in the IR spectra are the *ν* C=N bands at 1622 cm^−1^ (**4**) and 1627 cm^−1^ (**5**), respectively. These bands clearly indicate the transition from the delocalized amidinate NCN units in the salt-like amidinate precursors **2** and **3** (*ν* NCN 1523 cm^−1^ (**2**) and 1543 cm^−1^ (**3**)) to N–C=N moieties with localized carbon–nitrogen double and single bonds. Bands at 2590 cm^−1^ (**4**) and 2606 cm^−1^ (**5**) can be assigned to the B–H stretching vibrations, while typical Si–C stretch bands of the SiMe_3_ groups appear at *ν*_as_ 726 cm^−1^ and 750 cm^−1^ as well as *ν*_s_ 651 cm^−1^ and 658 cm^−1^, respectively. The ^1^H, ^13^C, and ^29^Si NMR spectra of **4** and **5** all showed only one singlet resonance for the SiMe_3_ groups. This is in agreement with the centrosymmetric molecular structure found in the X-ray structural analysis of **4** (see following paragraph). As was observed for the anionic precursors **2** and **3**, the ^11^B NMR spectra of the bis-silylated derivatives also displayed only single resonances around −13.4 ppm.

### 2.2. Crystal and Molecular Structures

**Figure 2:**
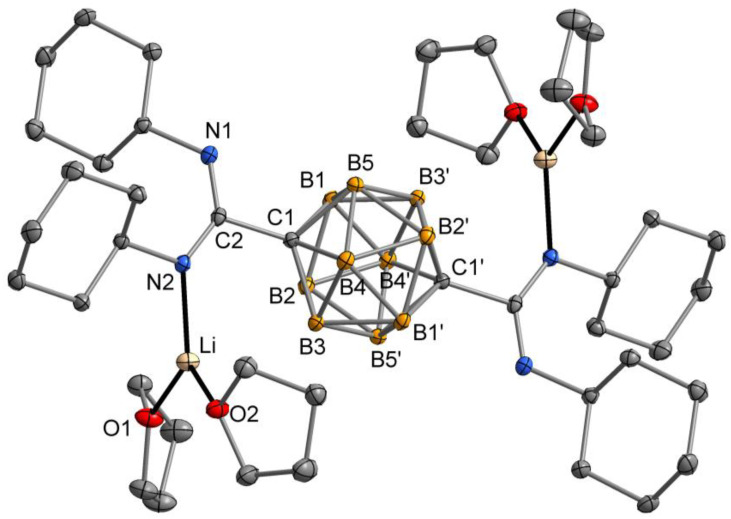
Molecular structure of **3** in the crystal. Displacement ellipsoids of the heavier atoms are drawn with 50% probability; H atoms are omitted for clarity. Selected bond lengths (Å) and angles (deg.): C(1)-C(2) 1.551(2), N(1)-C(2) 1.305(2), N(2)-C(2) 1.340(2), N(2)-Li 1.953(3), O(1)-Li 1.949(3), O(2)-Li 1.933(3), N(1)-C(2)-N(2) 136.9(1), N(1)-C(2)-C(1) 109.7(1), N(2)-C(2)-C(1) 113.4(1), C(2)-N(2)-Li 134.9(1). Symmetry code to generate equivalent atoms: ‘2–*x*, 1–*y*, 1–*z*.

**Figure 3:**
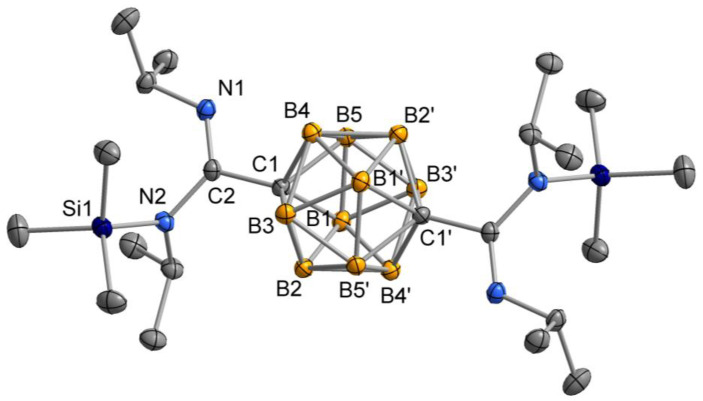
Molecular structure of **4** in the crystal. Displacement ellipsoids of the heavier atoms with 50% probability; H atoms are omitted for clarity. Selected bond lengths (Å) and angles (deg.): C(1)-C(2) 1.539(2), C(2)-N(1) 1.265(2), C(2)-N(2) 1.423(2), Si(1)-N(2) 1.751(1), N(1)-C(2)-N(2) 128.9(1), N(1)-C(2)-C(1) 113.8(1), N(2)-C(2)-C(1) 117.3(1), C(2)-N(2)-Si(1) 121.00(9). Symmetry code to generate equivalent atoms: ‘1–*x*,1–*y*, 1–*z*.

The title compounds **3** and **4** could be structurally characterized through X-ray diffraction studies. The molecular structures are depicted in Figure 2 and Figure 3. Colorless, prism-shaped single-crystals of **3** were grown from concentrated solutions in THF at r.t., while compound **4** was obtained in the form of well-formed, colorless, block-like single-crystals upon slow crystallization from toluene at 4 °C. As illustrated in Figure 2, structure determination of compound **3** confirmed the presence of an anionic *p*-carboranylamidinate species formed by the addition of dilithiated *p*-carborane to the central C atom of the carbodiimide reagent. The overall molecular structure is centrosymmetric. With 1.953(3) Å, the Li-N2 bond length is typical for a coordinative bond. As in other typical lithium amidinates such as Li[MeC_6_H_4_C(NSiMe_2_)_2_](THF)_2_ [36,47], the C–N distances in the amidinate NCN unit are quite similar (N(1)-C(2) 1.305(2), N(2)-C(2) 1.340(2)), indicating complete delocalization of the negative charge. However, the coordination of the lithium ion to the anionic amidinate moieties differs from the vast majority of other lithium amidinates in that it is not *κN*,*κN′*-chelating. Instead, the lithium ions are coordinated to only one nitrogen atom of the NCN moiety, resulting in a nearly trigonal planar coordination geometry around Li. There are only very few examples of similar monodentate amidinate coordination to lithium [48,49], and all of them result from steric crowding around the NCN unit, e.g., through very bulky terphenyl or triptycenyl substituents. Thus it can be assumed that steric congestion is also the reason for the rather unusual trigonal planar coordination of the lithium ions in compound **3**.

The neutral *p*-carboranyl-bis(amidine) derivative **4** crystallizes in the monoclinic space *P*2_1_/*n*, and, like **3**, the molecule also shows crystallographically imposed centrosymmetry. The transition from the delocalized anionic NCN moieties in the amidinate salts **2** and **3** to a neutral amidine is clearly evidenced by the change in the C–N bond lengths. With distances of C(2)-N(1) 1.265(2) Å and C(2)-N(2) 1.423(2) Å, compound **4** clearly contains N–C=N units with isolated single and double bonds. In this respect, the molecular structure of **4** is closely related to the oxygen analogue *p*-carborane-1,12-dicarboxylic acid [50]. The N1-C2-N2 angle is 128.9(1)°, and the Si-N2 distance is 1.751(1) Å. Both values are in good agreement with those of the silylated *o*-carboranylamidine derivative *o*-C_2_B_10_H_10_[*κC,N*-C(*^i^*PrNSiMe_3_)(=N*^i^*Pr)]SiMe_3_ [51].

## 3. Experimental Section

### 3.1. General Procedures and Instrumentation

All reactions were carried out in oven-dried or flame-dried glassware under an inert atmosphere of dry argon employing standard Schlenk and glovebox (MBraun MBLab) techniques. The solvents *n*-pentane, toluene, DME, and THF were distilled from sodium/benzophenone under nitrogen atmosphere prior to use. *p*-carborane was obtained from Katchem spol. s.r.o., 278 01 Kralupy nad Vltavou, Czech Republic (https://katchem.cz/en). Other starting materials were purchased from Sigma-Aldrich and used without further purification. All NMR spectra (^1^H, ^13^C, ^29^Si, ^11^B, and ^7^Li) were recorded in THF-*d*_8_ solutions on a Bruker DPX 400 spectrometer. IR spectra were measured with a Bruker Vertex 70V spectrometer equipped with a diamond ATR unit between 4000 cm^−1^ and 50 cm^−1^. Mass spectra were measured on a MAT 95 apparatus (EI, 70 eV). Microanalyses (C, H, N) were performed using a VARIO EL cube apparatus. Melting/decomposition points were determined using a Büchi Melting Point B-540.

### 3.2. Synthesis of Compound p-C_2_H_10_B_10_[C(N^i^Pr)_2_Li(DME)]_2_ *(**2**)*

A total of 0.50 g (3.5 mmol) *p*-carborane, dissolved in THF (50 mL), was treated at r.t. with 2 equiv. of *n*-butyllithium (7.0 mmol, 4.40 mL of a 1.6 M solution in *n*-hexane). After stirring for 1 h, 0.88 g (7.0 mmol) of 1,3-di-*iso*-propylcarbodiimide was added via syringe, and stirring at r.t. was continued for 12 h. The resulting clear yellow solution was evaporated to dryness and the oily crude product was redissolved in a minimum volume of DME (ca. 10 mL). Product **2** was precipitated by the addition of *n*-pentane (ca. 50 mL) and isolated after drying under vacuum as a microcrystalline, pale yellow solid in 51% isolated yield (1.06 g). M.p. 215 °C (dec.). Elemental analysis calcd. for C_24_H_58_B_10_N_4_O_4_Li_2_ (M = 588.71 g mol^−1^): C, 48.96%; H, 9.9%; N, 9.51%; found C, 48.91%; H, 9.77%; N, 9.64%. ^1^H NMR (400 MHz, THF-*d*_8_, 21 °C): δ = 3.53 (sept, 4 H, ^3^*J* = 6.40 Hz, CH-^i^Pr), 3.41, 3.25 (DME), 1.55–3.12 (m br, 10 H, BH), 0.78 (d, 24 H, ^3^*J* = 6.00 Hz, CH_3_-^i^Pr) ppm. ^13^C{^1^H} NMR (100.6 MHz, THF-*d*_8_, 23 °C): δ = 155.2 (NCN), 93.3 (C-NCN), 72.6, 58.9 (DME), 46.3 (CH-^i^Pr), 25.6 (CH_3_-^i^Pr) ppm. ^11^B{^1^H} NMR (128.4 MHz, THF-*d*_8_, 23 °C): δ = –14.1 ppm. ^7^Li{^1^H} NMR (155.5 MHz, THF-*d*_8_, 23 °C): δ = 0.11 ppm. IR (ATR): ν_max_ 2955 m (ν_s_ CH_3_), 2926 m (ν_as_ CH_2_), 2860 w (ν_as,s_ CH_3_/CH_2_), 2597 m (ν BH), 1523 s (ν NCN), 1461 m (δ_as,s_ CH_3_/CH_2_), 1367 m (δ_s_ CH_3_), 1357 m (δ_s_ CH_3_), 1312 m, 1285 m, 1264 s, 1191 w, 1157 w, 1112 m, 1077 vs (ν_as_ C-O-C), 1021 m, 961 w, 901 w, 870 m, 801 w, 737 m, 679 w, 621 m, 599 m, 566 m, 506 m, 494 m, 460 w, 385 w, 374 w, 242 vs cm^−1^. MS (EI, 70 eV): *m*/*z* (%) 396 (17) [((^i^PrN)_2_C)_2_-C_2_H_10_B_10_]^+^, 353 (27) [((^i^PrN)_2_C)_2_-C_2_H_10_B_10_-^i^Pr]^+^, 270 (15) [(^i^PrN)_2_C-C_2_H_10_B_10_]^+^, 227 (49) [(^i^PrN)NC-C_2_H_10_B_10_]^+^, 170 (25) [NC-C_2_H_10_B_10_]^+^, 143 (90) [C_2_H_9_B_10_]^+^, 58 (100) [^i^PrN + H]^+^.

### 3.3. Synthesis of Compound p-C_2_H_10_B_10_[C(NCy)_2_Li(THF)_2_]_2_ *(**3**)*

This compound was prepared in a similar manner as described for **2** but using 1.44 g (7.0 mmol) of *N,N’*-dicyclohexylcarbodiimide as a precursor. The resulting clear solution was concentrated to a total volume of ca. 30 mL which led to the formation of a white precipitate, which was then redissolved by brief heating. Colorless single crystals suitable for X-ray diffraction were obtained directly by storing the concentrated THF solution at r.t. for a few days. Yield: 1.37 g (46%). M.p. 238 °C (dec.). Elemental analysis calcd. for C_44_H_86_B_10_Li_2_N_4_O_4_ (M = 857.18 g mol^−1^): C, 61.65%; H, 10.11%; N, 6.54%; found C, 61.59%; H, 10.01%; N, 6.67%. ^1^H NMR (400 MHz, THF-*d*_8_, 24 °C): *δ* = 3.20–3.29 (m br, 2 H, C*H*-Cy), 3.60 (THF), 2.99–3.09 (m br, 2 H, C*H*-Cy), 1.50–2.90 (m br, 10 H, B*H*), 1.76 (THF), 1.63–1.73 (m, 10 H, C*H*_2_-Cy), 1.43–1.61 (m, 10 H, C*H*_2_-Cy), 1.12–1.34 (m, 20 H, C*H*_2_-Cy) ppm. ^13^C{^1^H} NMR (100.6 MHz, THF-*d*_8_, 24 °C): *δ* = 154.2, 154.0 (N*C*N), 68.1 (THF), 56.4, 56.1, 54.6, 54.4 (*C*H-Cy), 36.5, 35.8, 35.0, 34.9, 34.8, 34.7, 30.6, 27.8, 27.7, 27.0, 26.9, 26.4 (*C*H_2_-Cy), 26.3 (THF), 25.8, 25.7, 25.6, 25.4, 25.0, 24.9 (*C*H_2_-Cy) ppm. ^11^B{^1^H} NMR (128.4 MHz, THF-*d*_8_, 24 °C): *δ* = −14.0, −14.8, −15.8 ppm. ^7^Li{^1^H} NMR (MHz, THF-*d*_8_, 24 °C): *δ* = 0.09 ppm. IR (KBr disk): *ν*_max_ 3419 w, 3222 w, 3091 w, 2979 m, 2927 vs (*ν*_as_ CH_2_), 2852 s (*ν*_s_ CH_2_), 2611 m (*ν* BH), 2119 w, 1959 w, 1657 m, 1543 s (*ν* NCN), 1510 m, 1463 m, 1449 m (*δ*_s_ CH_2_), 1372 w, 1332 m, 1289 m, 1270 m, 1230 m, 1181 w, 1147 w, 1132 m, 1043 m (*ν*_as_ C-O-C), 970 w, 930 w, 888 m, 841 w, 833 w, 810 w, 794 w, 742 w, 699 w, 674 w, 589 w, 561 w, 511 w, 489 w, 477 w, 449 w, 437 w cm^−1^. MS (EI, 70 eV): *m*/*z* (%) 557 (45) [((CyN)_2_C)_2_C_2_H_10_B_10_ + H]^+^, 474 (27) [((CyN)_2_C)_2_C_2_H_10_B_10_ − C_6_H_11_ + H]^+^, 392 (100) [((CyN)_2_C)_2_C_2_H_10_B_10_ − 2C_6_H_11_ + 2H]^+^, 268 (33) [(CyN)NCC_2_H_10_B_10_ + H]^+^, 143 (16) [C_2_H_9_B_10_]^+^.

### 3.4. Synthesis of Compound p-C_2_H_10_B_10_[C(^i^PrN(SiMe_3_)(=N^i^Pr)]_2_ *(**4**)*

A solution of compound **2** (3.5 g in 50 mL) THF was prepared as described above and treated in situ with 0.90 mL (7.0 mmol) of chlorotrimethylsilane. After stirring for 12 h at r.t., the solvent was removed in vacuum, and the orange-yellow residue was extracted with toluene (50 mL) and filtered in order to remove the by-product LiCl. The concentration of the filtrate to a total volume of ca. 20 mL followed by cooling to 4 °C for several days led to the formation of colorless, block-like single crystals which were suitable for X-ray diffraction. Yield: 1.03 g (54%). M.p. 265 °C. Elemental analysis calcd. for C_22_H_54_B_10_N_4_Si_2_ (M = 538.98 g mol^−1^): C, 49.03%; H, 10.10%; N, 10.39%; found C, 48.82%; H, 10.54%; N, 10.50%. ^1^H NMR (400 MHz, THF-*d*_8_, 21 °C): *δ* = 3.60 (sept, 2 H, ^3^*J* = 6.00 Hz, C*H*-*^i^*Pr), 3.22 (m, 2 H, C*H*-*^i^*Pr), 1.70–3.10 (m br, 10 H, B*H*), 1.27 (d, 12 H, ^3^*J* = 5.60 Hz, C*H*_3_-*^i^*Pr), 0.97 (d, 12 H, ^3^*J* = 6.00 Hz, C*H*_3_-*^i^*Pr), 0.16 (s, 18 H, C*H*_3_-SiMe_3_) ppm. ^13^C{^1^H} NMR (100.6 MHz, THF-*d*_8_, 22 °C): *δ* = 152.4 (N*C*N), 89.5 (*C*-NCN), 51.1, 50.6 (*C*H-*^i^*Pr), 24.9, 23.6 (*C*H_3_-*^i^*Pr), 3.82 (*C*H_3_-SiMe_3_) ppm. ^11^B{^1^H} NMR (128.4 MHz, THF-*d*_8_, 22 °C): *δ* = –13.4 ppm. ^29^Si{^1^H} NMR (79.5 MHz, THF-*d*_8_, 21 °C): *δ* = –1.40 ppm. IR (ATR): *ν*_max_ 2987 w (*ν*_s_ CH_3_), 2966 m, 2932 w, 2889 w (*ν*_as_ CH_3_), 2641 w, 2623 m, 2590 m (*ν* BH), 1622 m (*ν* C=N), 1468 w (*δ*_as_ CH_3_), 1450 w, 1405 w, 1375 m (*δ*_s_ CH_3_), 1362 w (*δ*_s_ CH_3_), 1319 m, 1255 m, 1207 s, 1159 m, 1139 m, 1117 m, 1092 m, 1042 w, 1008 m, 979 m, 930 w, 887 m, 858 m, 833 vs (*ρ* CH_3_), 750 m, 726 m (*ν*_as_ SiC_3_), 678 m, 651 m (*ν*_s_ SiC_3_), 623 w, 607 w, 582 w, 548 w, 516 m, 471 m, 423 w, 379 w, 333 w, 260 m, 176 w, 140 w, 87 w, 68 w, 61 m cm^−1^. MS (EI, 70 eV): *m*/*z* (%) 541 (3) [M]^+^, 526 (5) [M − CH_3_]^+^, 498 (100) [M − 3CH_3_]^+^, 468 (29) [M − Si(CH_3_)_3_]^+^, 342 (16) [(*^i^*PrN)(*^i^*PrNSiMe_3_)CC_2_H_10_B_10_]^+^.

### 3.5. Synthesis of Compound p-C_2_H_10_B_10_[C(CyN(SiMe_3_)(=NCy)]_2_ *(**5**)*

Compound **5** was prepared in the same manner as described above using 3.00 g (3.5 mmol) of **3** and 0.90 mL (7.0 mmol) chlorotrimethylsilane to afford colorless crystals after crystallization from a small amount of toluene at −32 °C. Yield: 1.05 g (43%). M.p. 159 °C. Elemental analysis calcd. for C_34_H_72_B_10_N_4_Si_2_ (M = 701.25 g mol^−1^): C, 59.23%; H, 10.35%; N, 7.99%; found C, 58.20%; H, 10.28%; N, 7.94%. ^1^H NMR (400 MHz, THF-*d*_8_, 38 °C): *δ* = 3.32–3.36 (m, 2 H, C*H*-Cy), 1.90–3.15 (m br, 10 H, B*H*), 2.73–2.79 (m, 2 H, C*H*-Cy), 1.04–1.86 (m, 24 H, C*H*_2_-Cy), 0.15 (s, 18 H, C*H*_3_-SiMe_3_) ppm. ^13^C{^1^H} NMR (100.6 MHz, THF-*d*_8_, 23 °C): *δ* = 152.0 (N*C*N), 89.6 (*C*-NCN), 60.1, 58.8 (*C*H-Cy), 35.8, 35.2, 35.1, 33.6. 27.6, 27.0, 26.8, 26.4, 24.6, 24.5 (*C*H_2_-Cy), 4.0 (*C*H_3_-SiMe_3_) ppm. ^11^B{^1^H} NMR (128.4 MHz, THF-*d*_8_, 23 °C): *δ* = –13.4 ppm. ^29^Si{^1^H} NMR (MHz, THF-*d*_8_, 22 °C): *δ* = 0.20 ppm. IR (ATR): *ν*_max_ 2930 m (*ν*_as_ CH_2_), 2852 m (*ν*_as,s_ CH_3_/CH_2_), 2644 s, 2606 m (*ν* BH), 2119 m, 1658 w, 1627 m (*ν* C=N), 1495 w, 1450 m (*δ*_as,s_ CH_3_/CH_2_), 1407 w, 1386 w (*δ*_s_ CH_3_), 1358 w, 1344 w, 1297 w, 1251 m, 1189 s, 1179 m, 1142 m, 1115 m, 1076 m, 1028 m, 996 m, 981 m, 956 m, 890 m, 857 m, 831 vs (*ρ* CH_3_), 820 vs, 777 m, 750 m (*ν*_as_ SiC_3_), 730 m, 701 m, 673 m, 658 m (*ν*_s_ SiC_3_), 630 m, 564 w, 540 w, 520 w, 497 w, 474 m, 461 m, 409 m, 384 m, 341 m, 322 m, 291 m, 260 m, 207 w, 181 w, 162 w, 136 w, 117 w, 86 w, 69 w, 57 m cm^−1^. MS (EI, 70 eV): *m*/*z* (%) 702 (3) [M]^+^, 687 (3) [M − CH_3_]^+^, 629 (32) [M − Si(CH_3_)_3_]^+^, 619 (71) [M − C_6_H_11_]^+^, 604 (12) [M − C_6_H_11_ − CH_3_]^+^, 556 (2) [M − 2 Si(CH_3_)_3_]^+^, 530 (67) [M − Si(CH_3_)_3_ − C_6_H_11_ − CH_3_]^+^, 279 (75) [CyN(Si(CH_3_)_3_)CNCy]^+^, 83 (100) [C_6_H_11_]^+^.

### 3.6. X-ray Crystallography

Single-crystal X-ray intensity data of **3** and **4** were collected on a STOE IPDS 2T diffractometer [52] equipped with a 34 cm image plate detector, using graphite-monochromated Mo-K_α_ radiation. The structure was solved by dual-space methods (SHELXT-2014/5) [53] and refined by full-matrix least-squares methods on *F*^2^ using SHELXL-2017/1 [54]. Crystallographic data for the title compounds have been deposited at the CCDC, 12 Union Road, Cambridge CB21EZ, U.K. Copies of the data can be obtained free of charge via the depository numbers 2248726 (**3**) and 2248725 (**4**) (e-mail: deposit@ccdc.cam.ac.uk, http://www.ccdc.cam.ac.uk).

## 4. Conclusions and Future Outlook

In summary, we succeeded in the synthesis and full characterization of the first amidinate and amidine derivatives of *para*-carborane. Lithium carboranylamidinates based on *p*-carborane are readily accessible by the addition of in situ-prepared 1,12-dilithio-*p*-carborane to 1,3-diorganocarbodiimides, R–N=C=N–R (R = *^i^*Pr, Cy). This result showed that all three isomers of C_2_B_10_H_12_ react in completely different manners with carbodiimides. An initial reactivity study involving treatment of **2** and **3** with 2 equiv. of Me_3_SiCl revealed that neutral bis-silylated amidine derivatives are also easily prepared. It should be noted here that the oxygen analogue *p*-carborane-1,12-dicarboxylic acid has been successfully utilized as a linker in the design of carborane-based MOFs (=metal-organic frameworks) [55,56]. One could easily foresee that the amidinate and amidine derivatives of *p*-carborane reported here will play a similar fruitful role in MOF chemistry in the future.

## Data Availability

Not applicable.

## Supplementary Materials

The following supporting information can be downloaded at: https://www.mdpi.com/article/10.3390/molecules28093837/s1: IR, NMR, and mass spectra for all title compounds as well as X-ray diffraction data for **3** and **4**.

## Sample Availability

Samples of the compounds are not available from the authors.
