# Supplementary material for: Synthesis and Structural Characterization of p-Carboranylamidine Derivatives
Source: Molecules. 2023 Apr 30;28(9):3837. doi: 10.3390/molecules28093837 (PMC10179778; doi:10.3390/molecules28093837)
Supplement: Supplementary file 1 [file molecules-28-03837-s001.zip › molecules-2360310-supplementary.pdf]

Supporting Information

Synthesis and structure of the first p-carboranylamidine derivatives

Nicole Harmgarth[1], Phil Liebing[2], Volker Lorenz[1], Felix Engelhardt[2], Liane Hilfert[1], Sabine Busse[1], Rüdiger Goldhahn[3] and Frank T. Edelmann[3,*]

---

[a] Dr. N. Harmgarth, Dr. P. Liebing, Dr. V. Lorenz, Dr. F. Engelhardt, Dr. L. Hilfert, Dr. S. Busse
Institut für Chemie
Otto-von-Guericke-Universität Magdeburg
Universitätsplatz 2, 39106 Magdeburg, Germany

[b] Dr. P. Liebing
Institut für Anorganische und Analytische Chemie
Friedrich-Schiller-Universität Jena
Humboldtstr. 8, 07743 Jena, Germany

[c] Prof. Dr. R. Goldhahn, Prof. Dr. F. T. Edelmann
Institut für Physik
Otto-von-Guericke-Universität Magdeburg
Universitätsplatz 2, 39106 Magdeburg, Germany

Table of content

		page
I.	Spectroscopic methods	S3
II.	IR spectra	S4
III.	NMR spectra	S6
IV.	Mass spectra	S14
V.	Single-crystal X-ray diffraction data	S18

I. Spectroscopic methods

IR spectra were measured with a Bruker Vertex 70V spectrometer equipped with a diamond ATR unit between 4000 cm^{-1} and 50 cm^{-1}. All NMR spectra (^{1}H, ^{13}C, ^{29}Si, ^{11}B, and ^{7}Li) were recorded in THF-d_8 solutions on a Bruker DPX 400 spectrometer. Mass spectra were measured on a MAT 95 apparatus (EI, 70 eV).

II. IR spectra

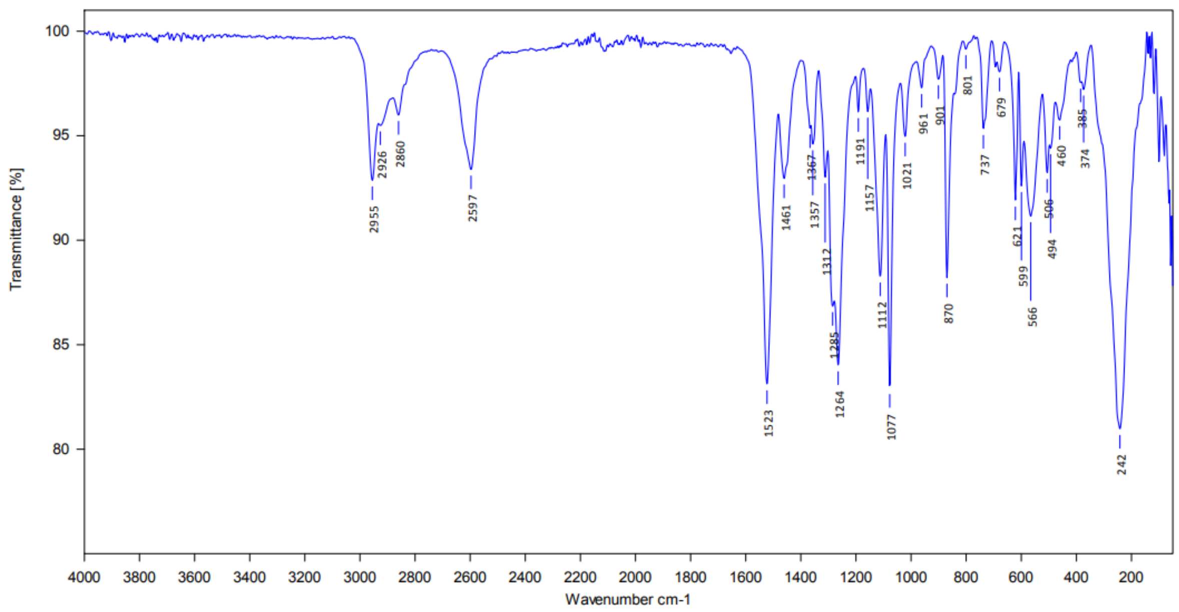

Fig. S1: IR spectrum of compound **2**

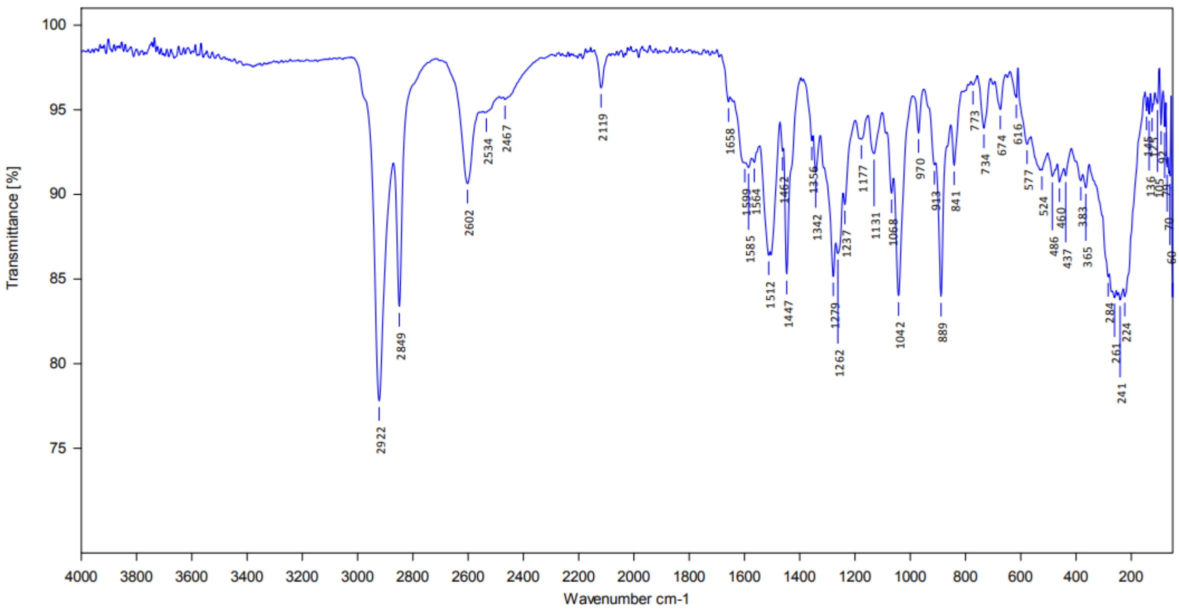

Fig. S2: IR spectrum of compound **3**

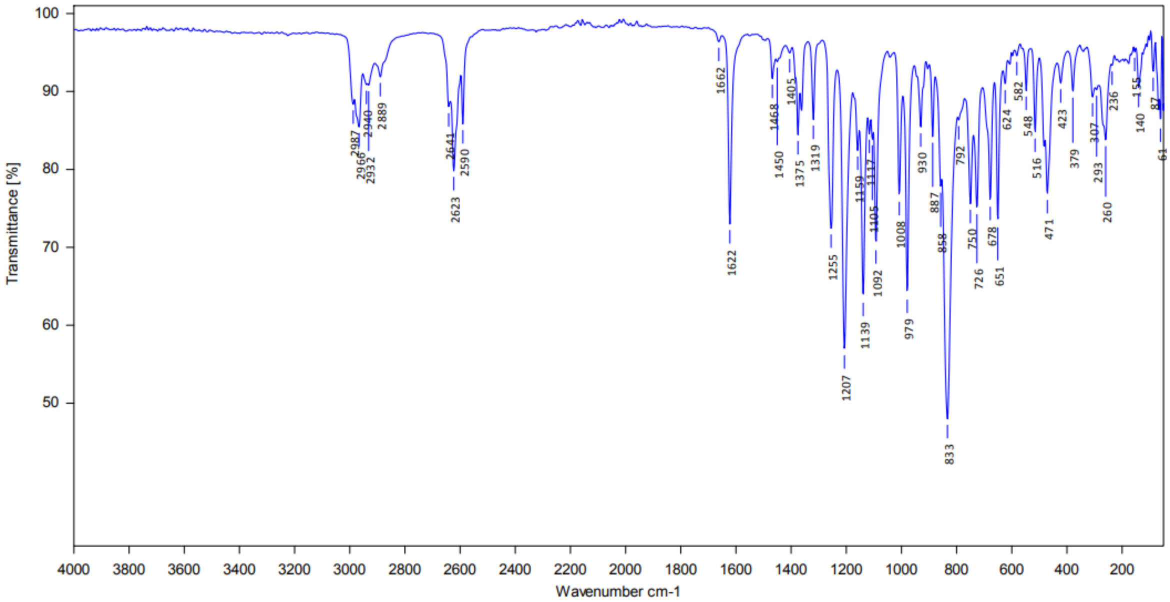

Fig. S3: IR spectrum of compound **4**

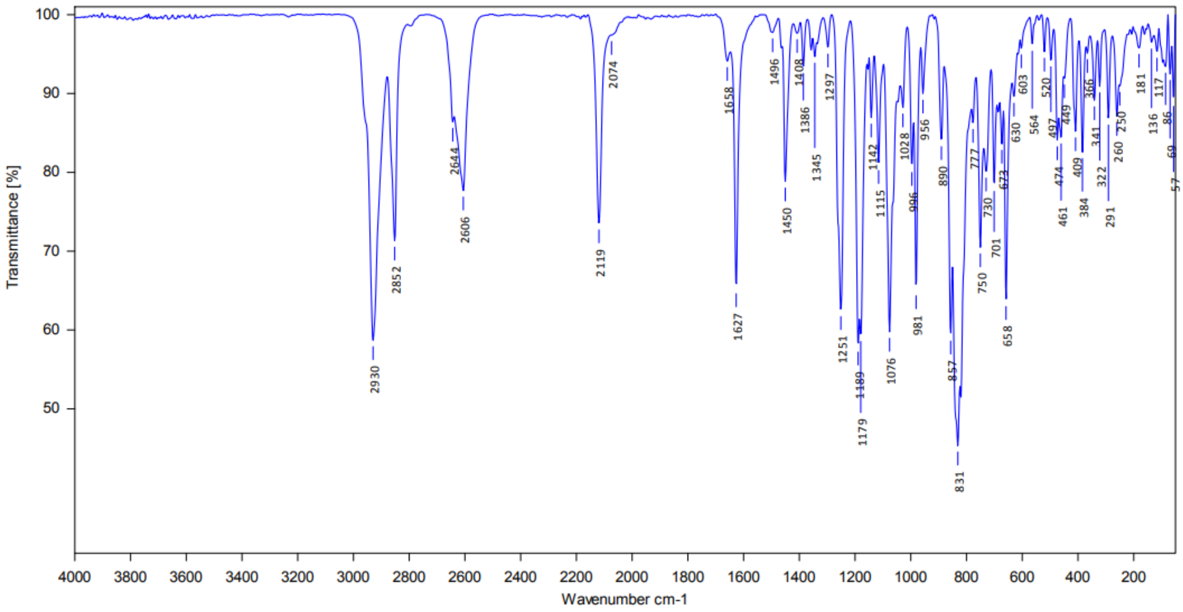

Fig. S4: IR spectrum of compound **5**

III. NMR spectra

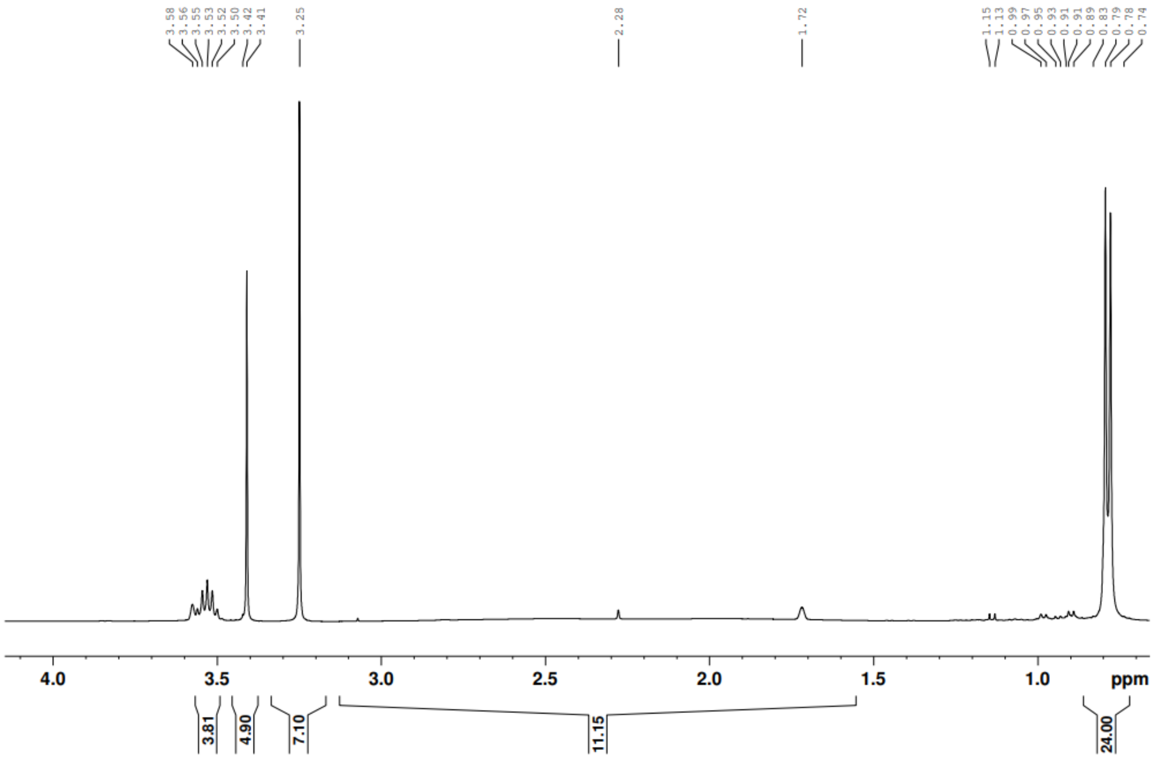

Fig. S5: ^{1}H NMR spectrum of compound **2**

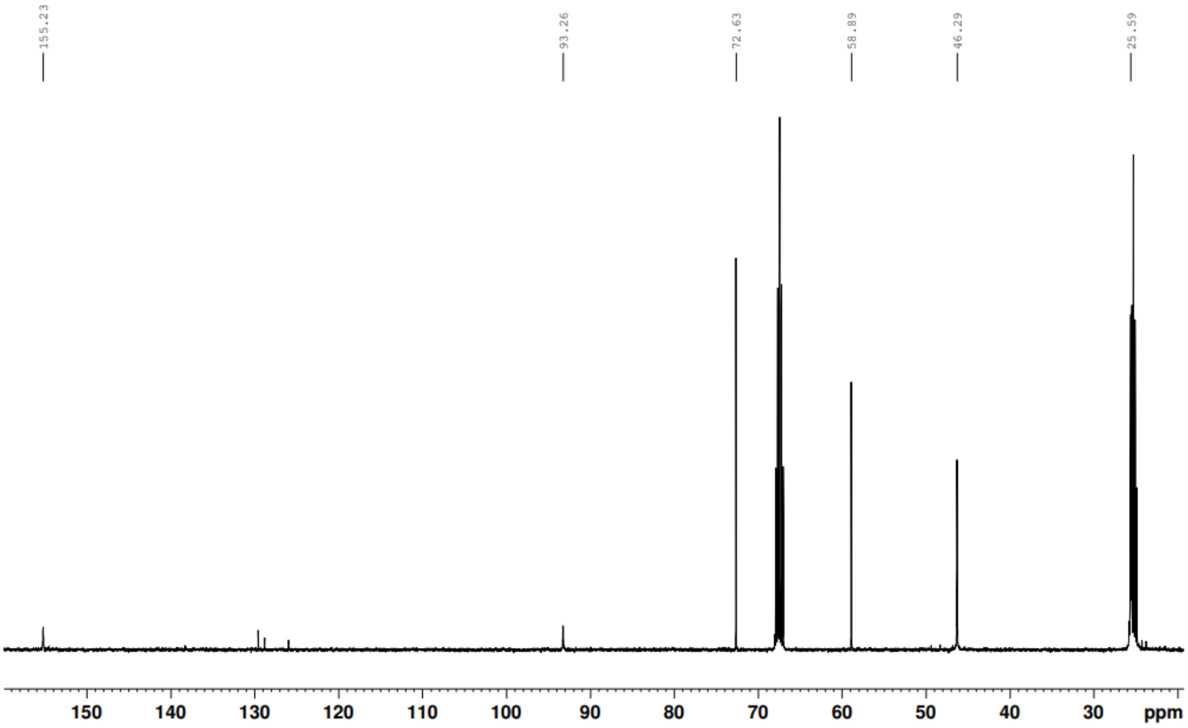

Fig. S6: ^{13}C NMR spectrum of compound **2**

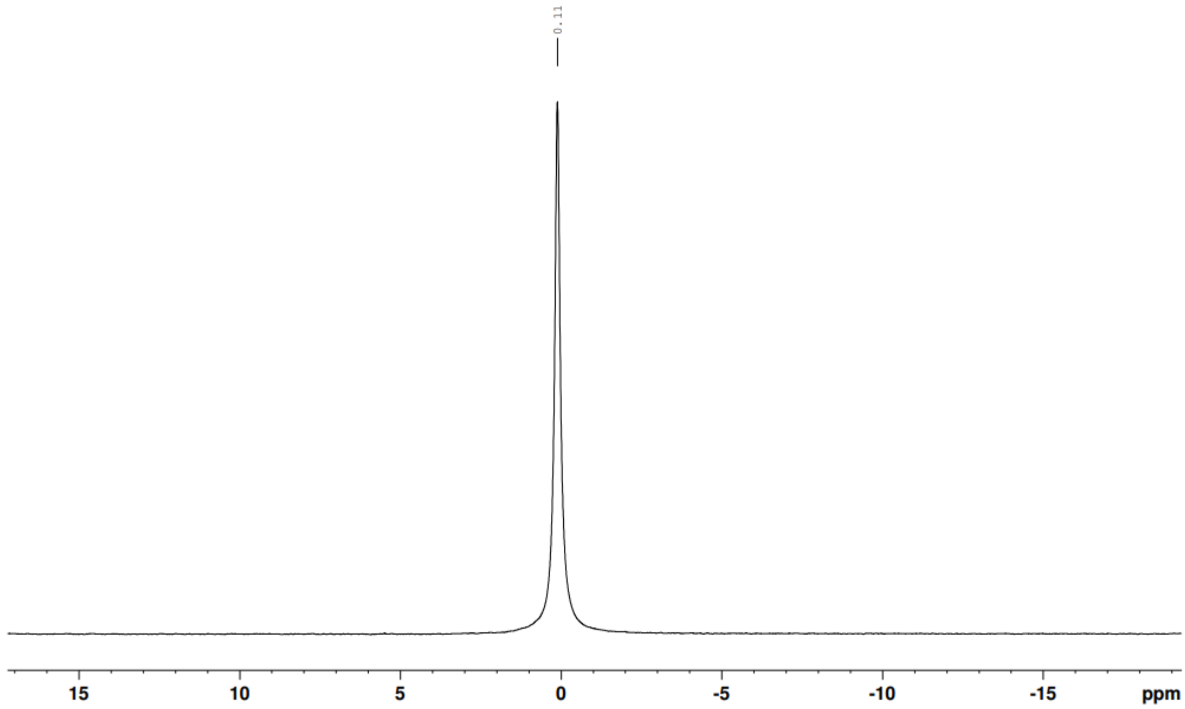

Fig. S7: ^{7}Li NMR spectrum of compound **2**

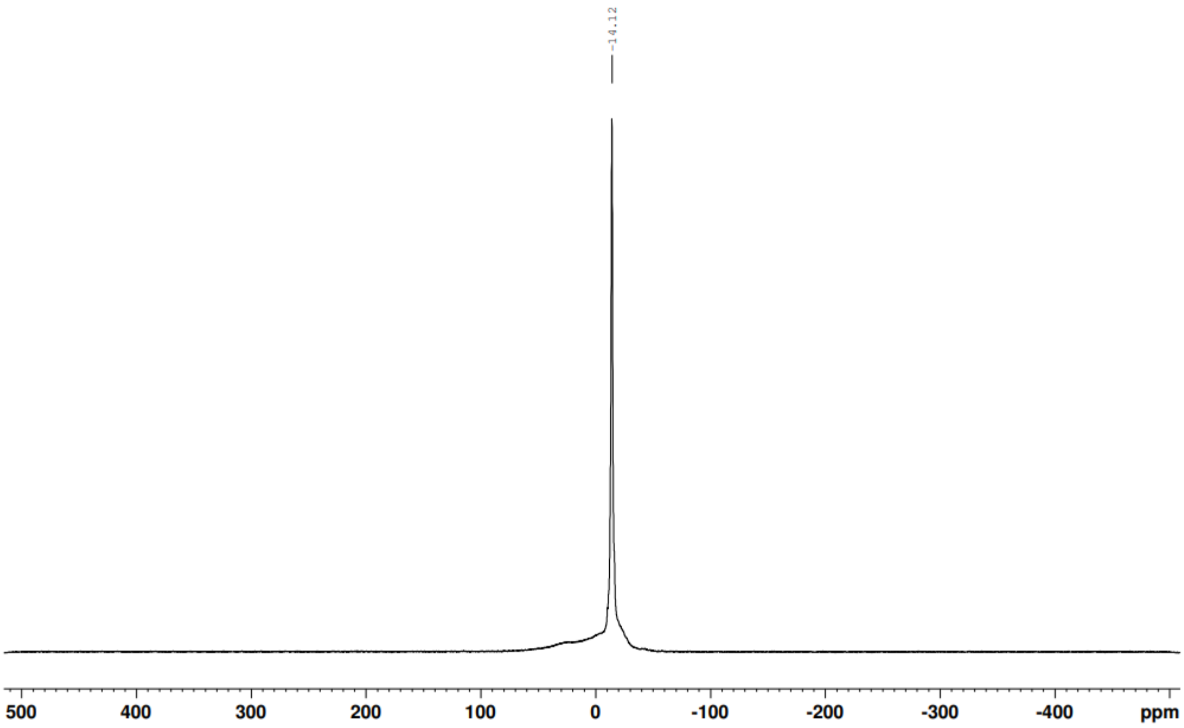

Fig. S8: ^{11}B NMR spectrum of compound **2**

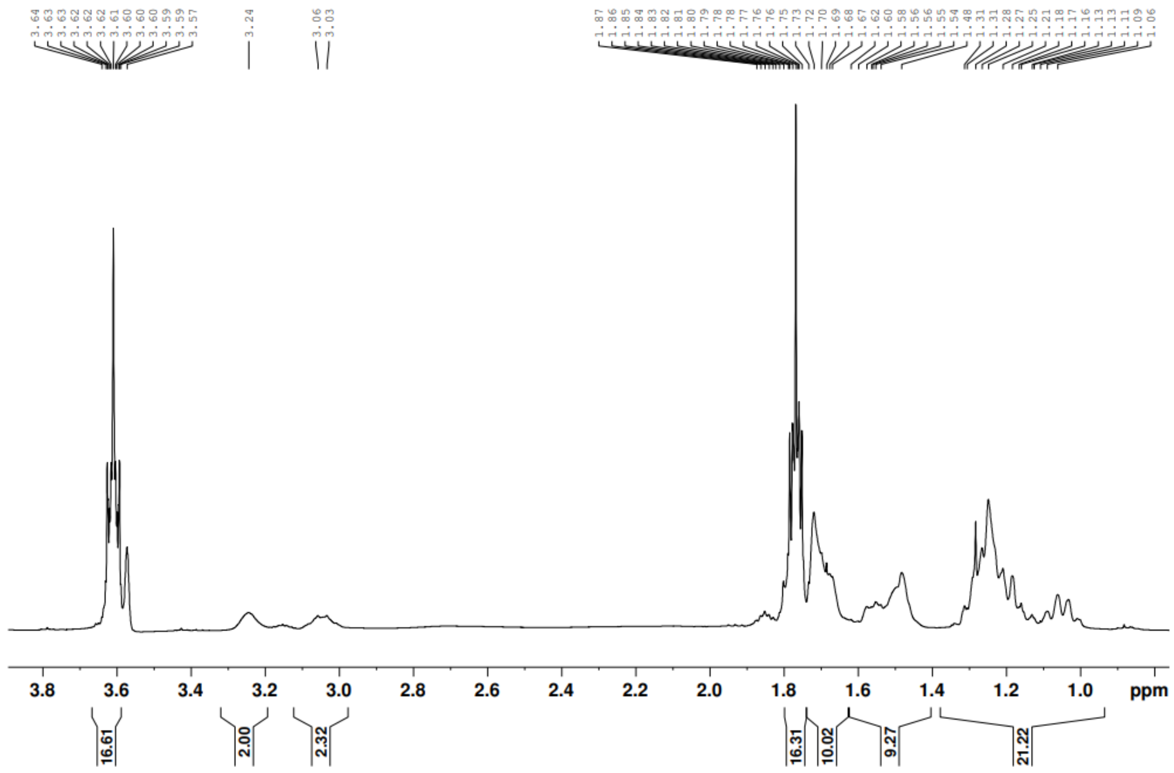

Fig. S9: ^{1}H NMR spectrum of compound **3**

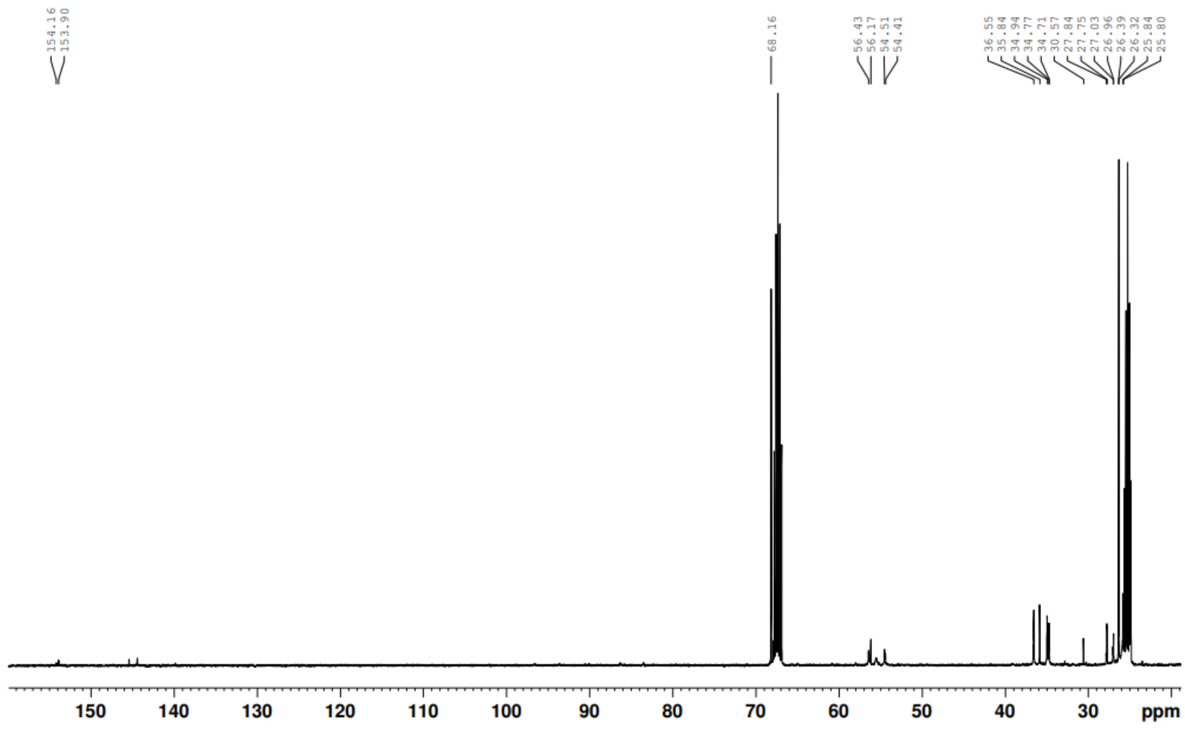

Fig. S10: ^{13}C NMR spectrum of compound **3**

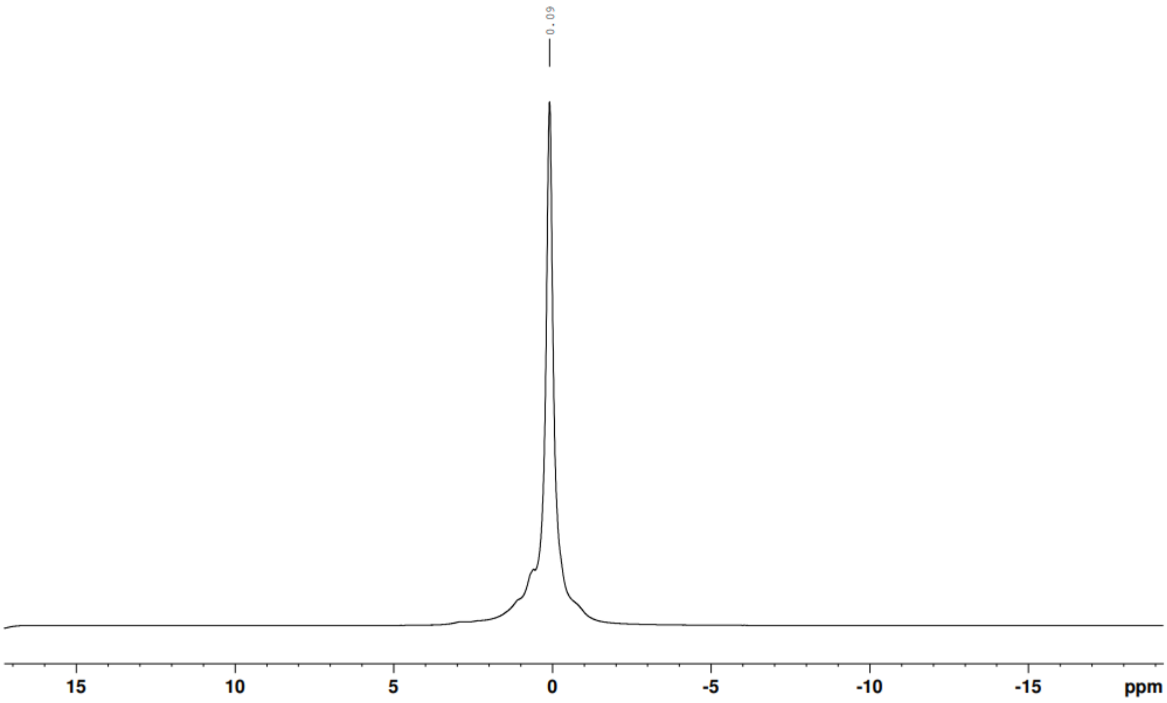

Fig. S11: ^{7}Li NMR spectrum of compound **3**

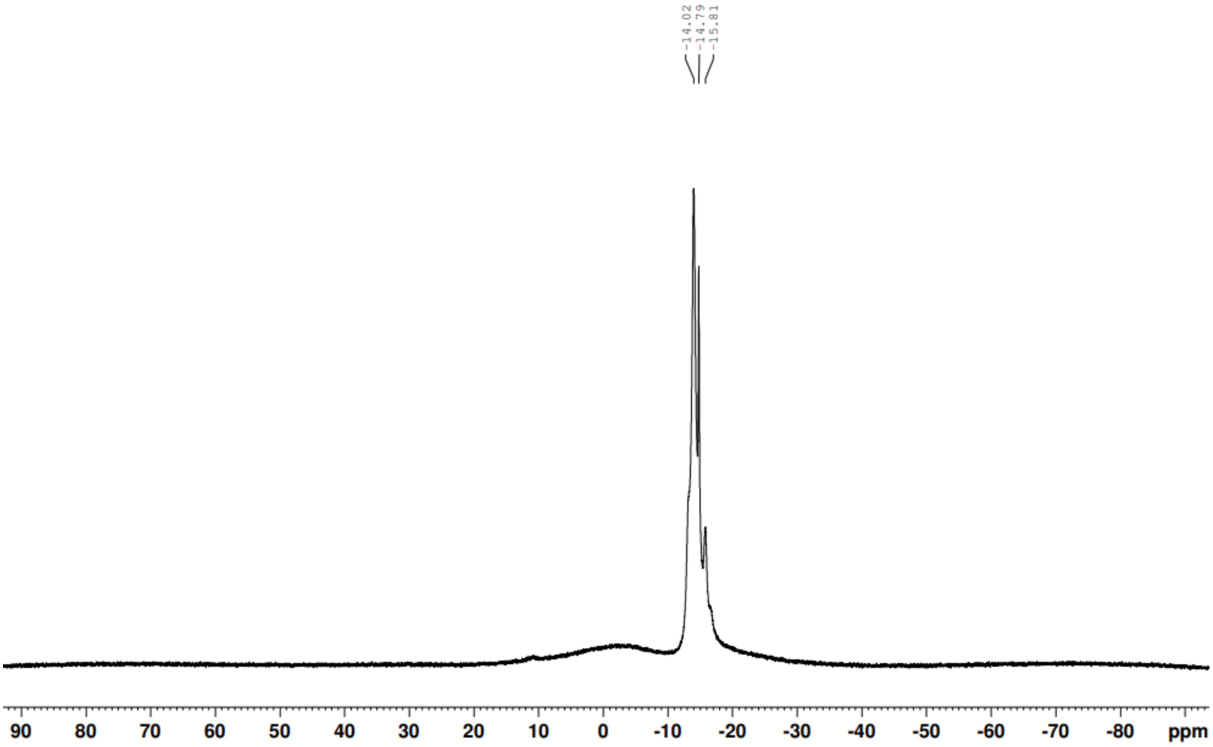

Fig. S12: ^{11}B NMR spectrum of compound **2**

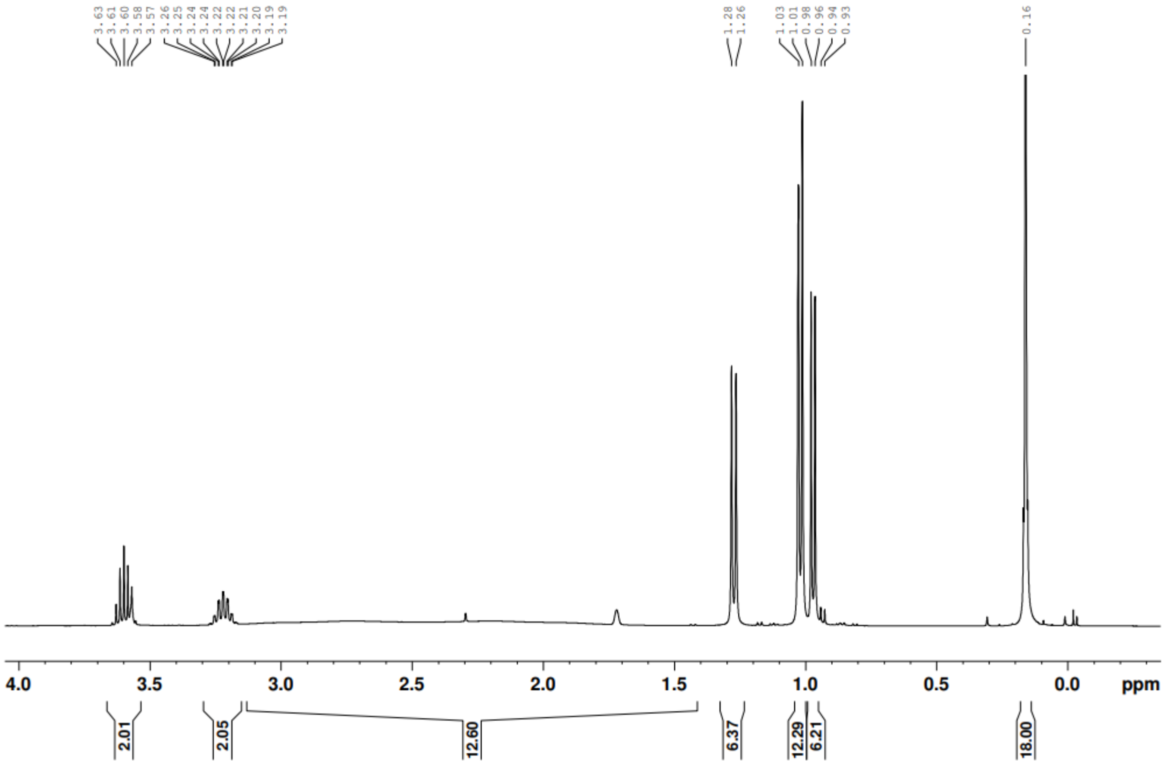

Fig. S13: ^{1}H NMR spectrum of compound **4**

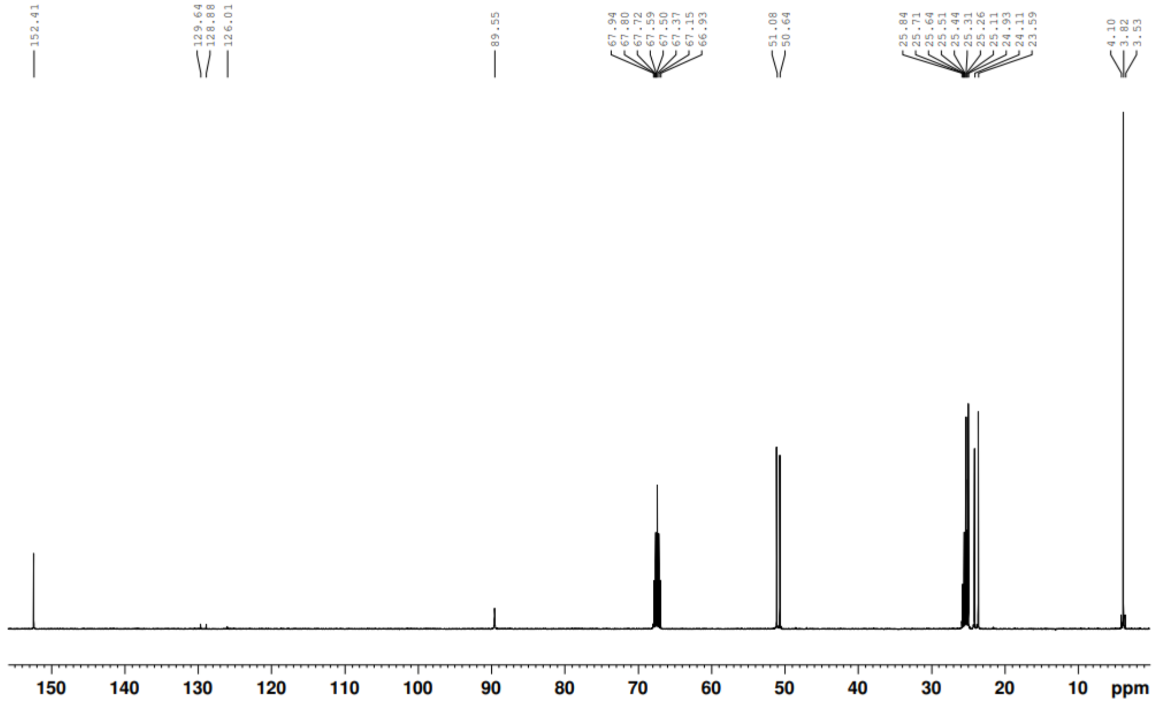

Fig. S14: ^{13}C NMR spectrum of compound **4**

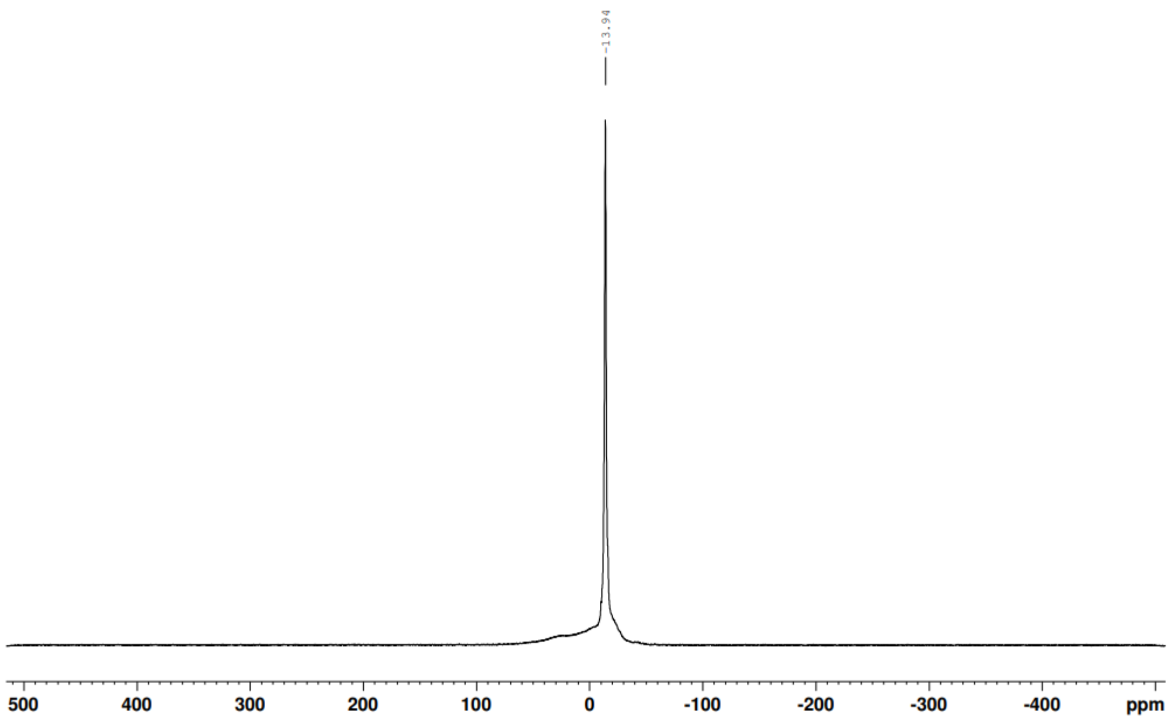

Fig. S15: ^{11}B NMR spectrum of compound **4**

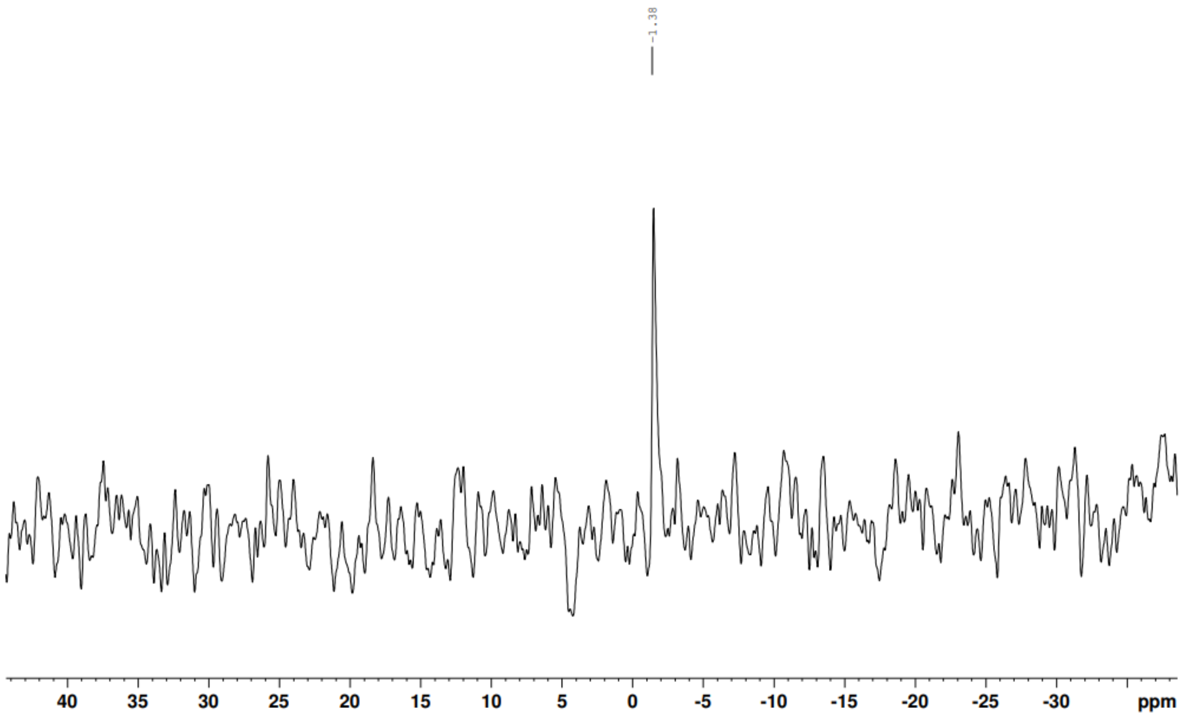

Fig. S16: ^{29}Si NMR spectrum of compound **5**

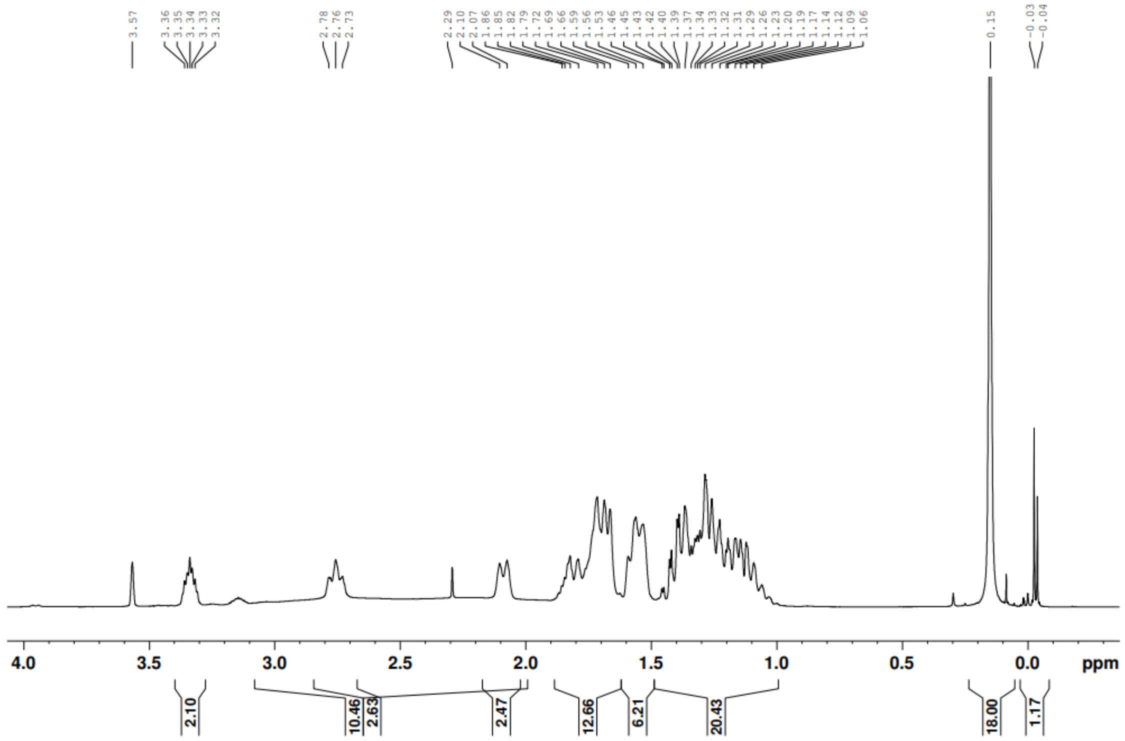

Fig. S17: ^{1}H NMR spectrum of compound **5**

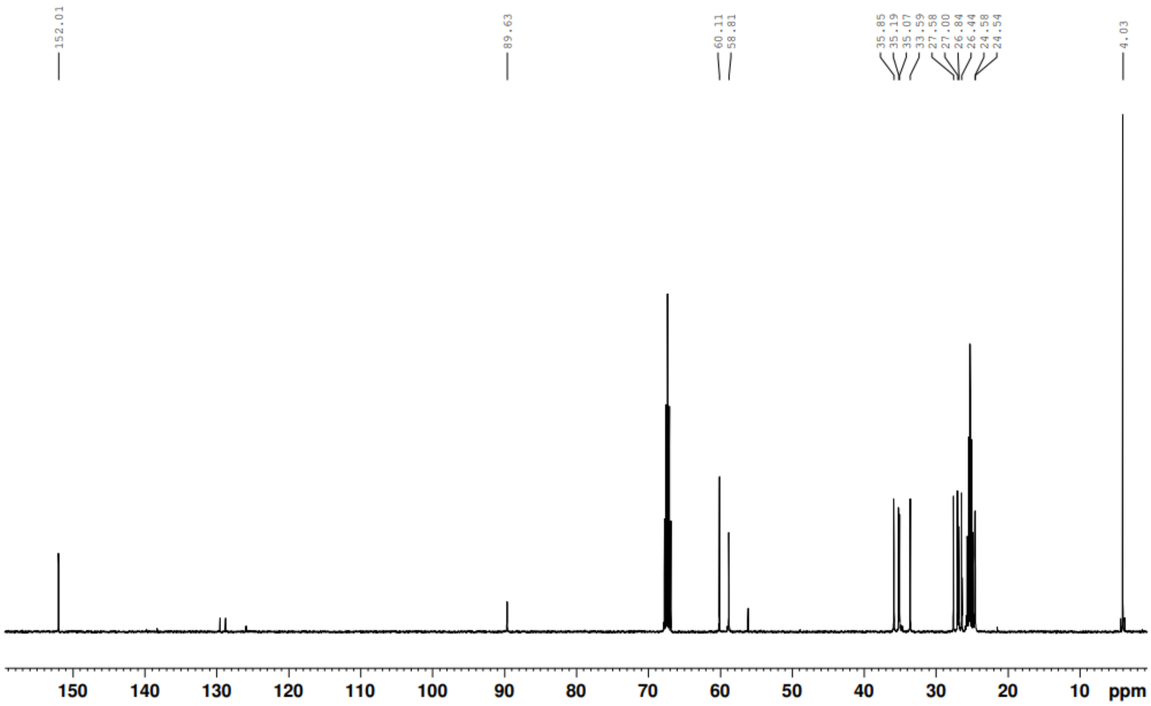

Fig. S18: ^{13}C NMR spectrum of compound **5**

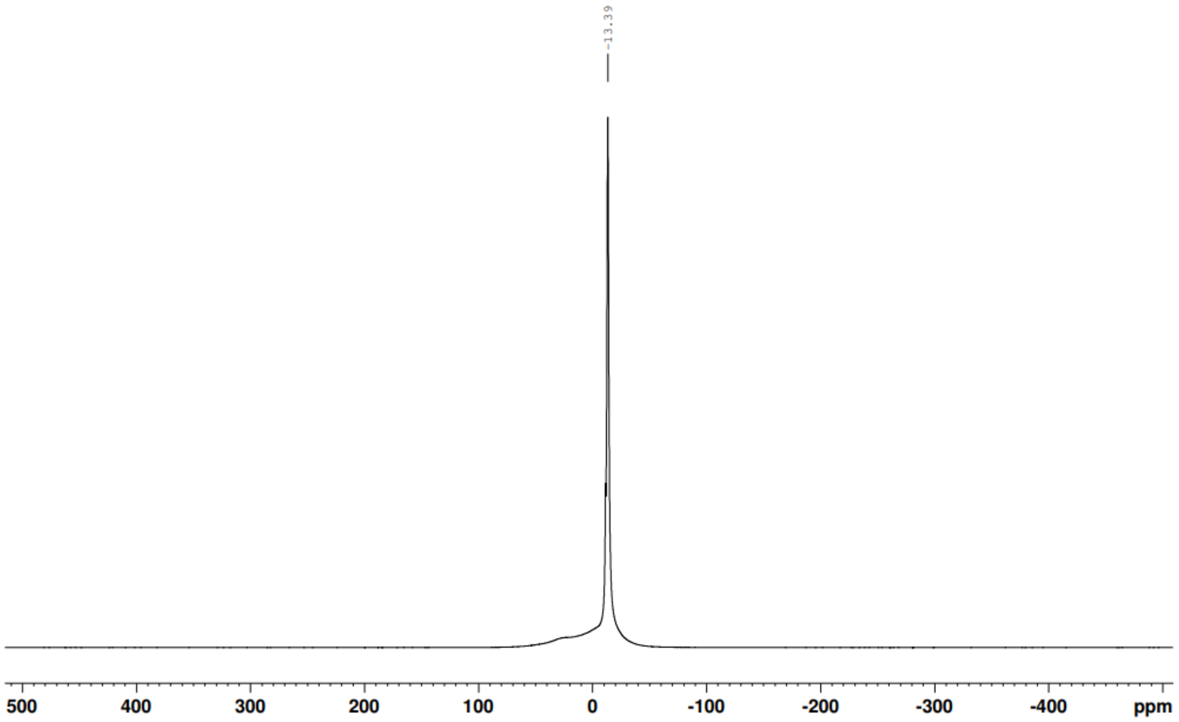

Fig. S19: ^{11}B NMR spectrum of compound **5**

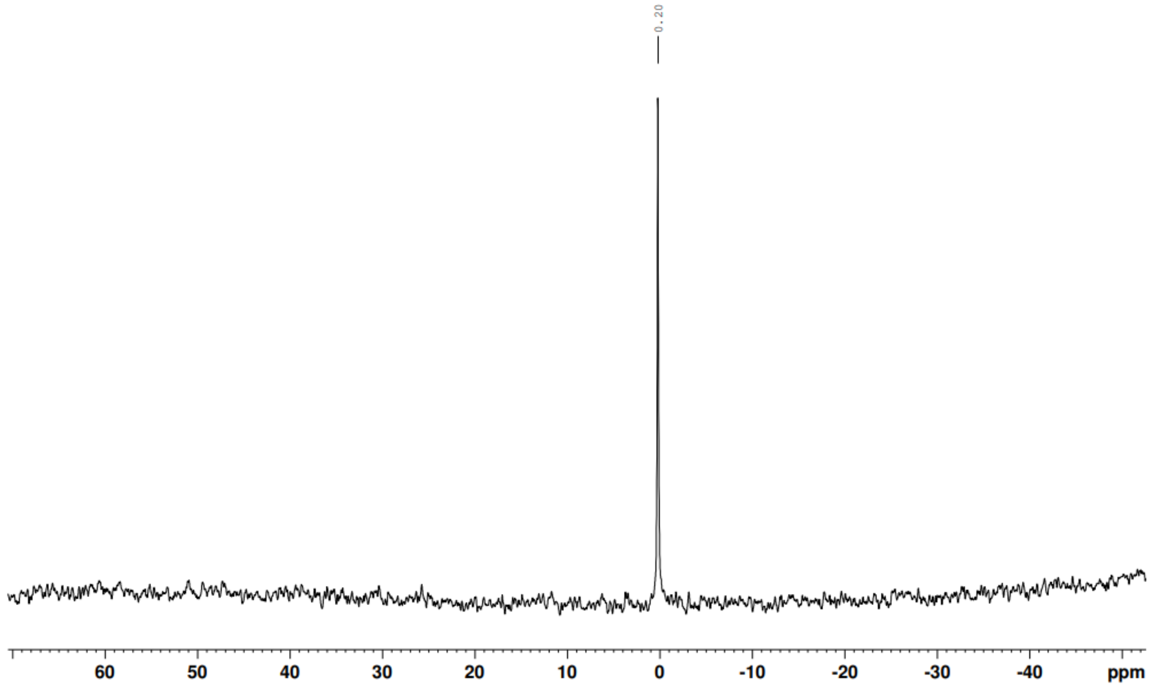

Fig. S20: ^{29}Si NMR spectrum of compound **5**

IV. Mass spectra

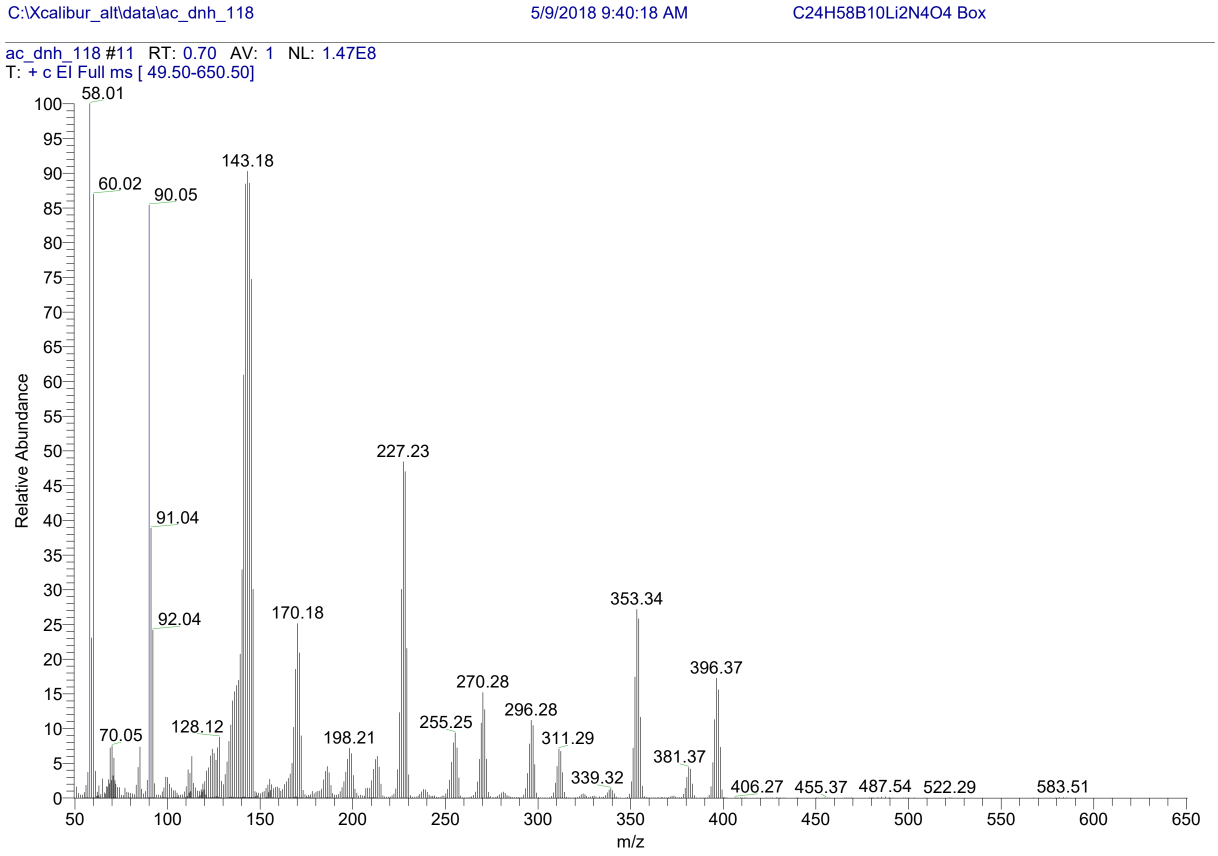

Fig. S21. Mass spectrum of compound **2**

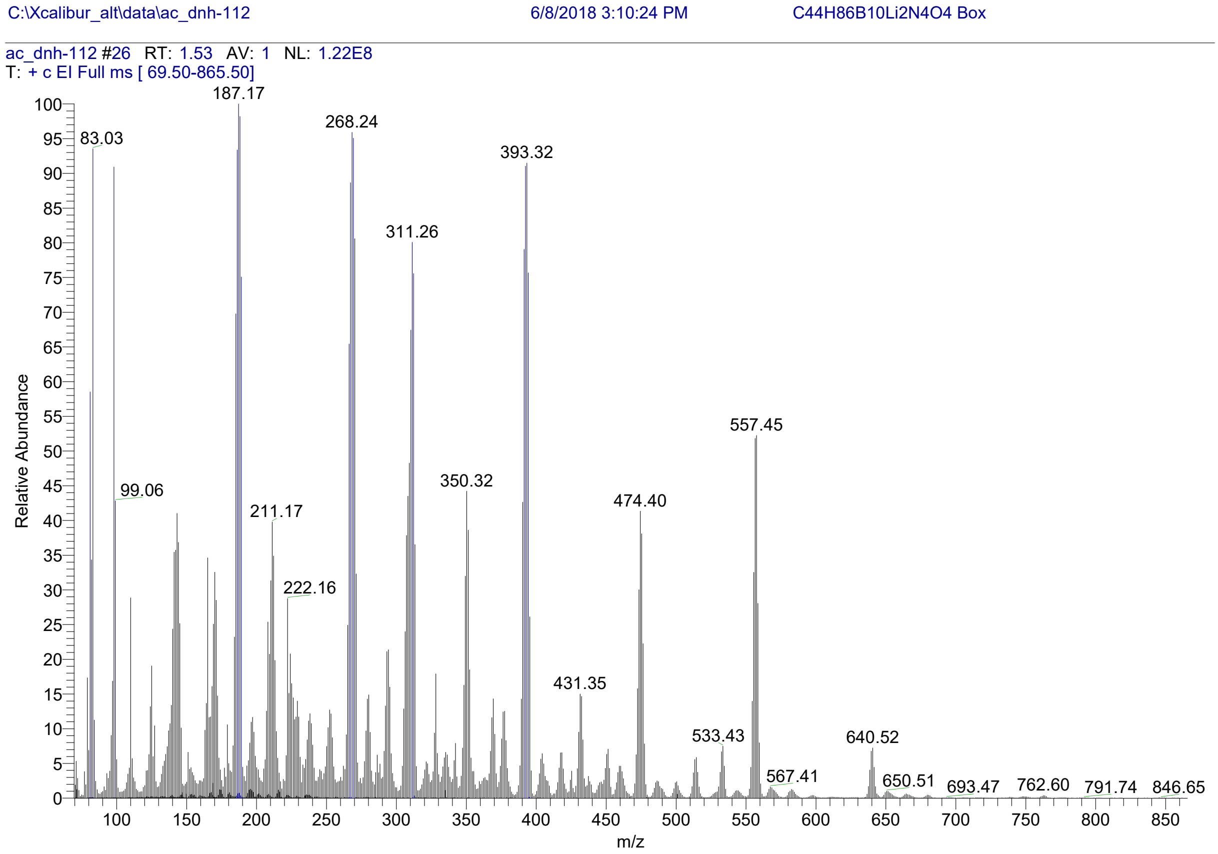

Fig. S22. Mass spectrum of compound **3**

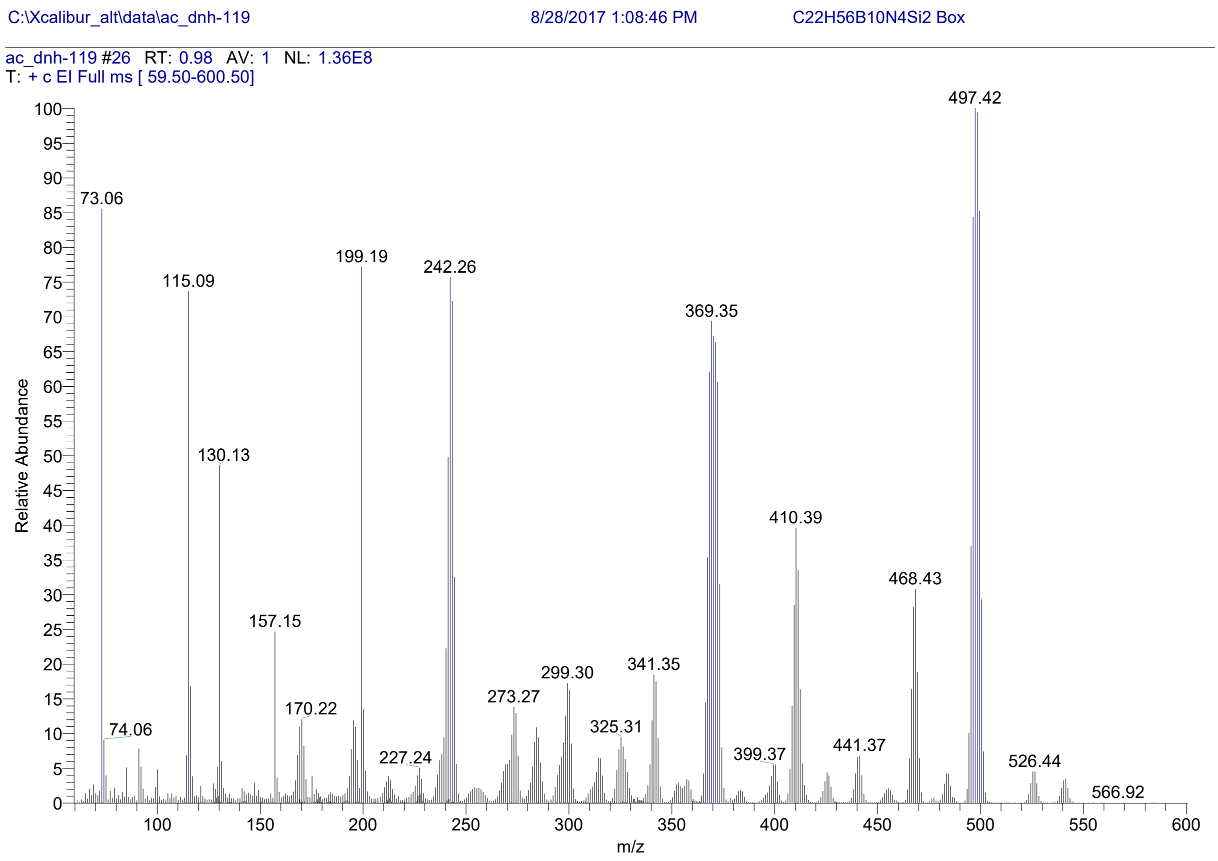

Fig. S23. Mass spectrum of compound **4**

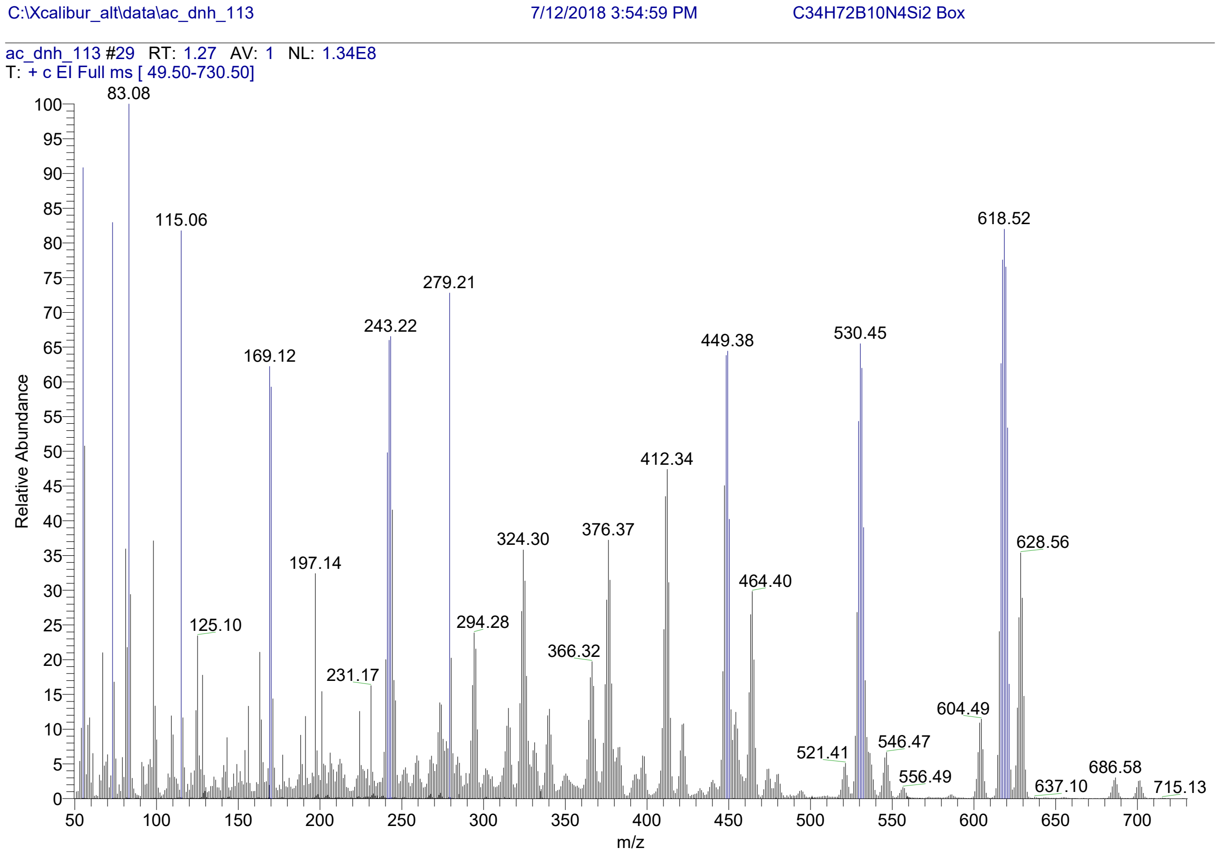

Fig. S24. Mass spectrum of compound **5**

V. Single-crystal X-ray diffraction data

Crystallographic Data and Details on Structure Refinement of Compound 3

formula sum	$C_{44}H_{86}B_{10}Li_2N_4O_4$
formula weight	857.14
crystal size (mm)	0.18 x 0.33 x 0.48 mm
crystal system	triclinic
space group	$P\overline{1}$
unit cell parameters	
a (Å)	8.809(2)
b (Å)	10.149(2)
c (Å)	14.728(3)
α (deg)	89.48(3)
β (deg)	81.54(3)
γ (deg)	70.59(3)
unit cell volume V ($Å^3$)	1227.2(5)
molecules per cell z	1
crystallographic density *μ* calcd (g cm^{-3})	1.160
absorption coefficient *μ* (mm^{-1})	0.068
diffractometer	STOE IPDS 2T
radiation (λ [Å])	graphite-monochromated Mo-K_{α} (0.71073)
temperature (°C)	–173(2)
scan type	ω scan (increment 1.5°, exposure 1 min)
completeness of dataset	0.992%
θ range of data collection (deg)	2.481 to 26.000
reflections collected	10451
independent reflections	4797
independent reflections with I>2σ(I)	4015
structure solution method	dual-space structure solution (SHELXT)
refinement method	full-matrix least-squares on F^2 (SHELXL)
absorption correction method	none
data / parameters / restraints	4797 / 294 / 0
goodness of fit (GooF) [all data]	1.027
final R values	
R_1 [all data, I ≥ 2σ (I)]	0.0554, 0.0430
wR_2 [all data, I ≥ 2σ (I)]]	0.1083, 0.1030
largest difference peak and hole	0.445 and –0.171 e$Å^{-3}$

Crystallographic Data and Details on Structure Refinement of Compound 4

formula sum	$C_{22}H_{54}B_{10}N_4Si_2$
formula weight	538.98
crystal size (mm)	0.20 x 0.20 x 0.20 mm
crystal system	Monoclinic
space group	P 2_1/n
unit cell parameters	
a (Å)	8.246(5)
b (Å)	14.278(4)
c (Å)	14.194(6)
α (deg)	90
β (deg)	98.29(4)
γ (deg)	90
unit cell volume *V* ($Å^3$)	1653(1)
molecules per cell *z*	4
crystallographic density μ_{calcd} (g cm^{-3})	1.086
absorption coefficient μ (mm^{-1})	0.127
diffractometer	STOE IPDS 2T
radiation (λ [Å])	graphite-monochromated Mo-K_α (0.71073)
temperature (°C)	–173(2)
scan type	ω scan (increment 1.5°, exposure 15 min)
completeness of dataset	99.3%
ϑ range of data collection (deg)	2.034 to 29.145
reflections collected	22162
independent reflections	4427
independent reflections with I>2σ(I)	4037
structure solution method	dual-space structure solution (SHELXT)
refinement method	full-matrix least-squares on F^2 (SHELXL)
absorption correction method	none
data / restraints / parameters	4427 / 160 / 179
goodness of fit (GooF) [all data]	1.179
final *R* values	
R_1 [all data, $I \geq 2\sigma(I)$]	0.0516, 0.0452
wR_2 [all data, $I \geq 2\sigma(I)$]]	0.1104, 0.1075
largest difference peak and hole	0.351 and -0.245 e$Å^{-3}$
